# Supplementary material for: Influences of age and gender on operative risks following carotid endarterectomy: A systematic review and meta-analysis
Source: PLoS One. 2023 May 10;18(5):e0285540. doi: 10.1371/journal.pone.0285540 (PMC10171679; doi:10.1371/journal.pone.0285540)

S1 Fig. Other main results

S1.1 Fig. 30 days combined stroke and death risk between age ≥75 years vs <75 years

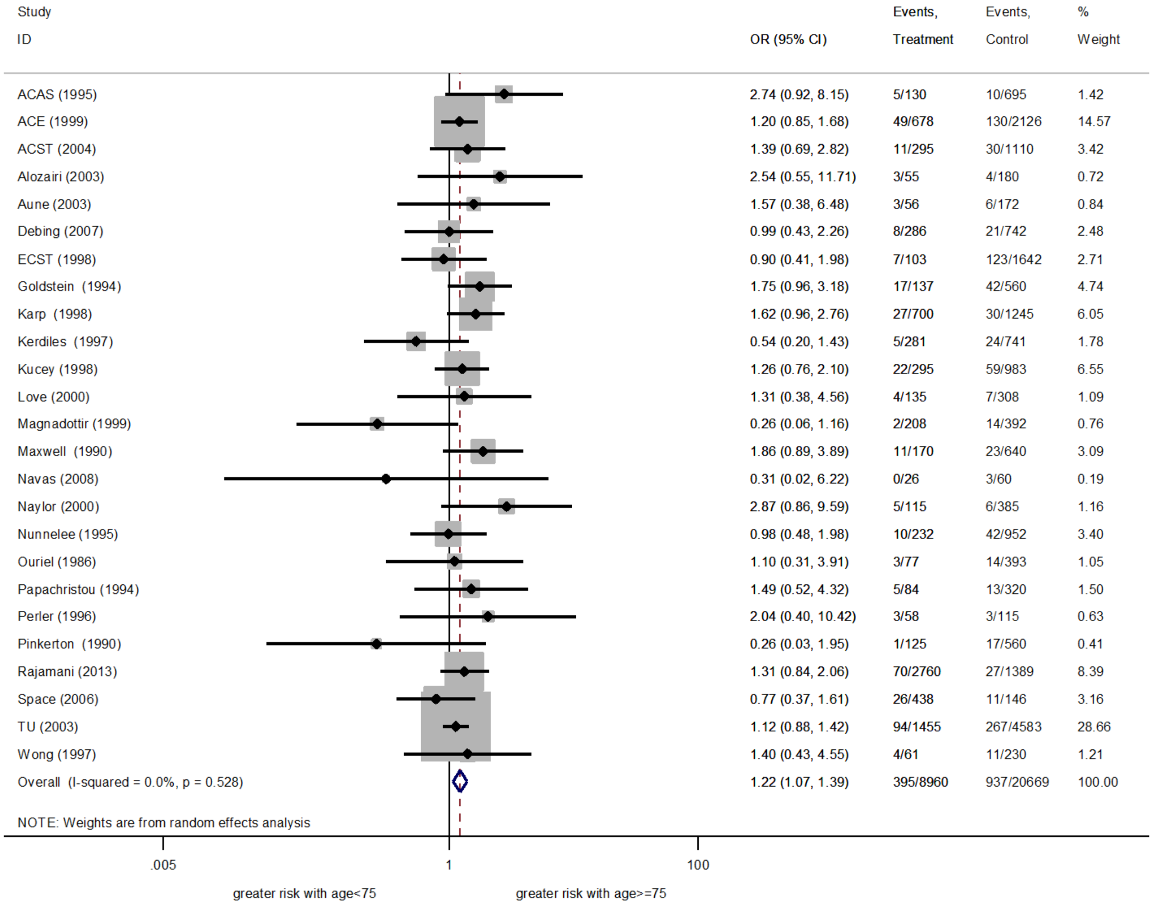

S1.2 Fig. 30 days death risk between age ≥80 years vs <80 years

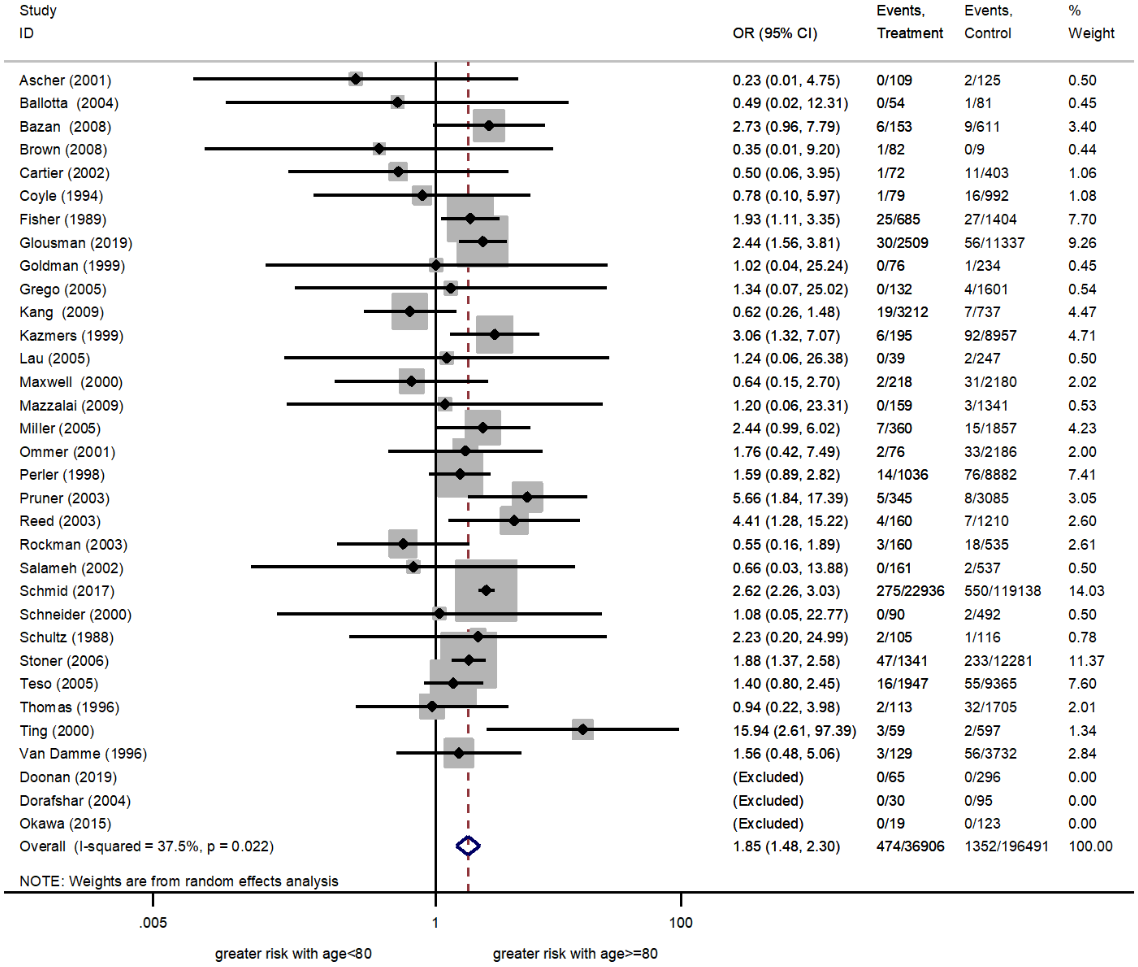

S1.3 Fig. 30 days combined stroke and death risk between age ≥80 years vs <80 years

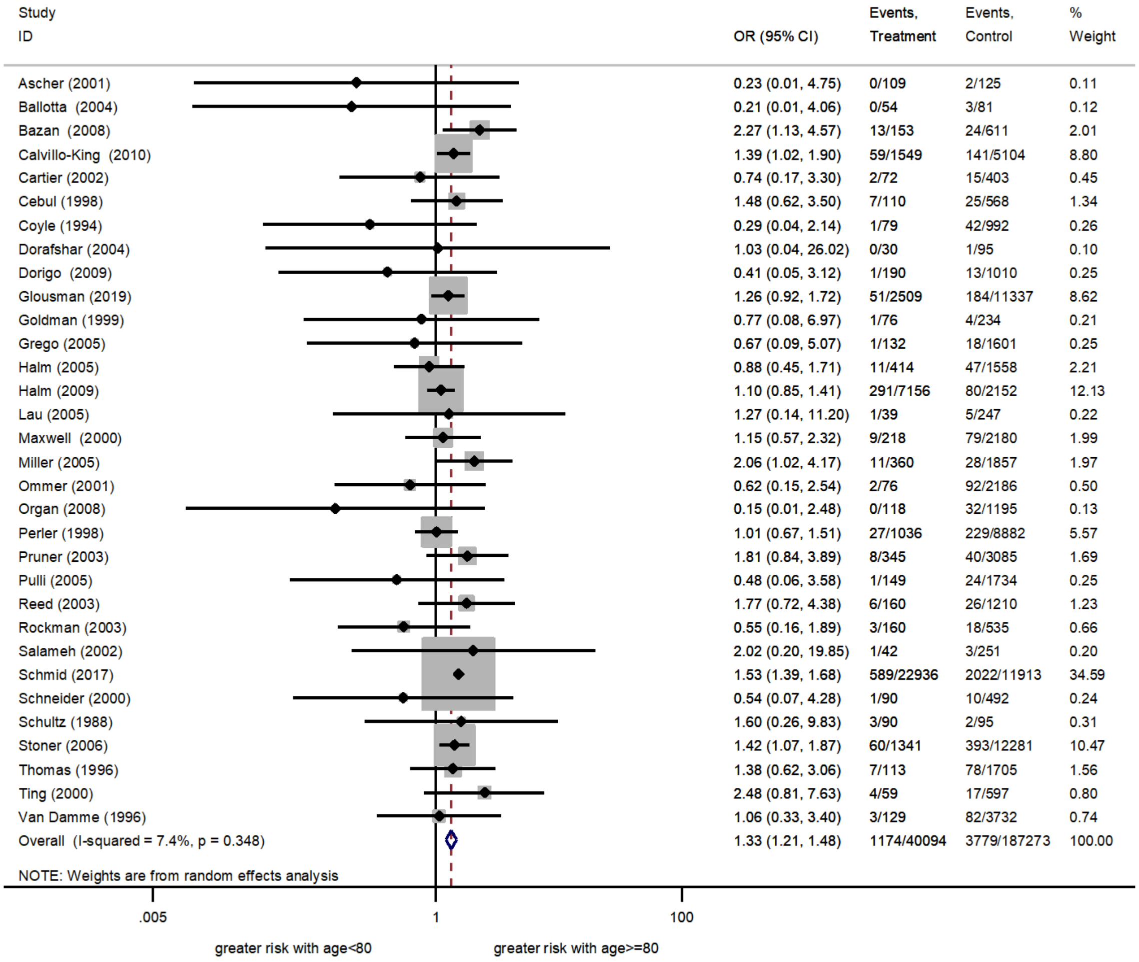

S1.4 Fig. 30 days stroke risk between age ≥75 years vs <75 years

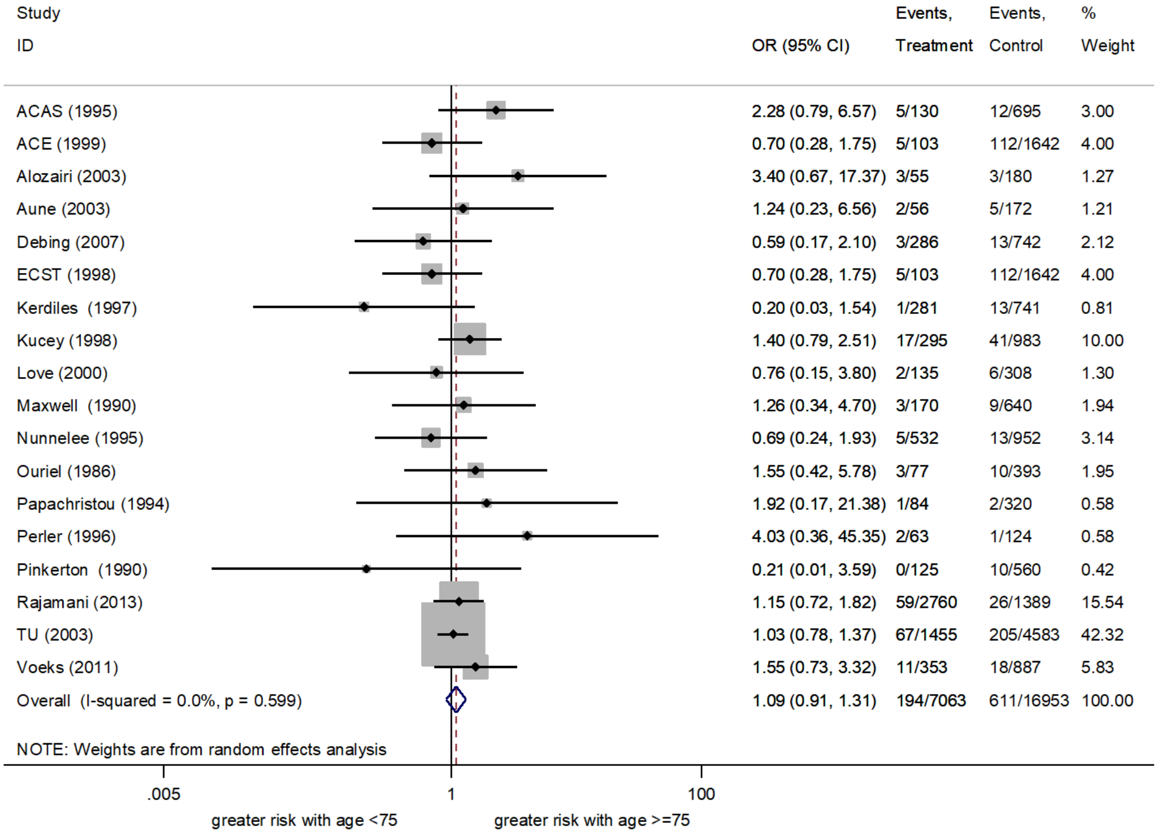

S1.5 Fig. 30 days combined stroke and death risk between female vs male

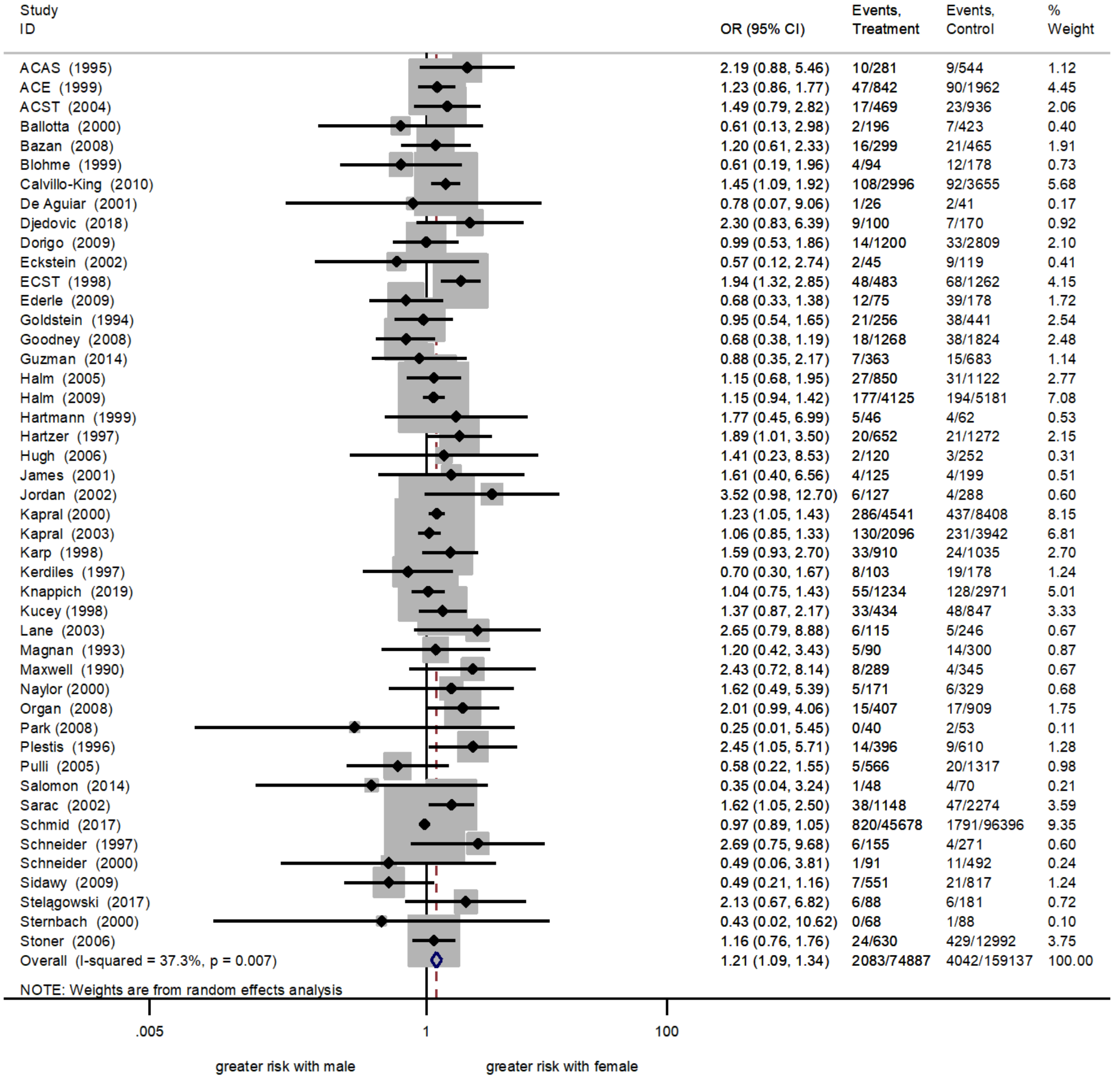

S1.6 Fig: 30 days death risk between female vs male

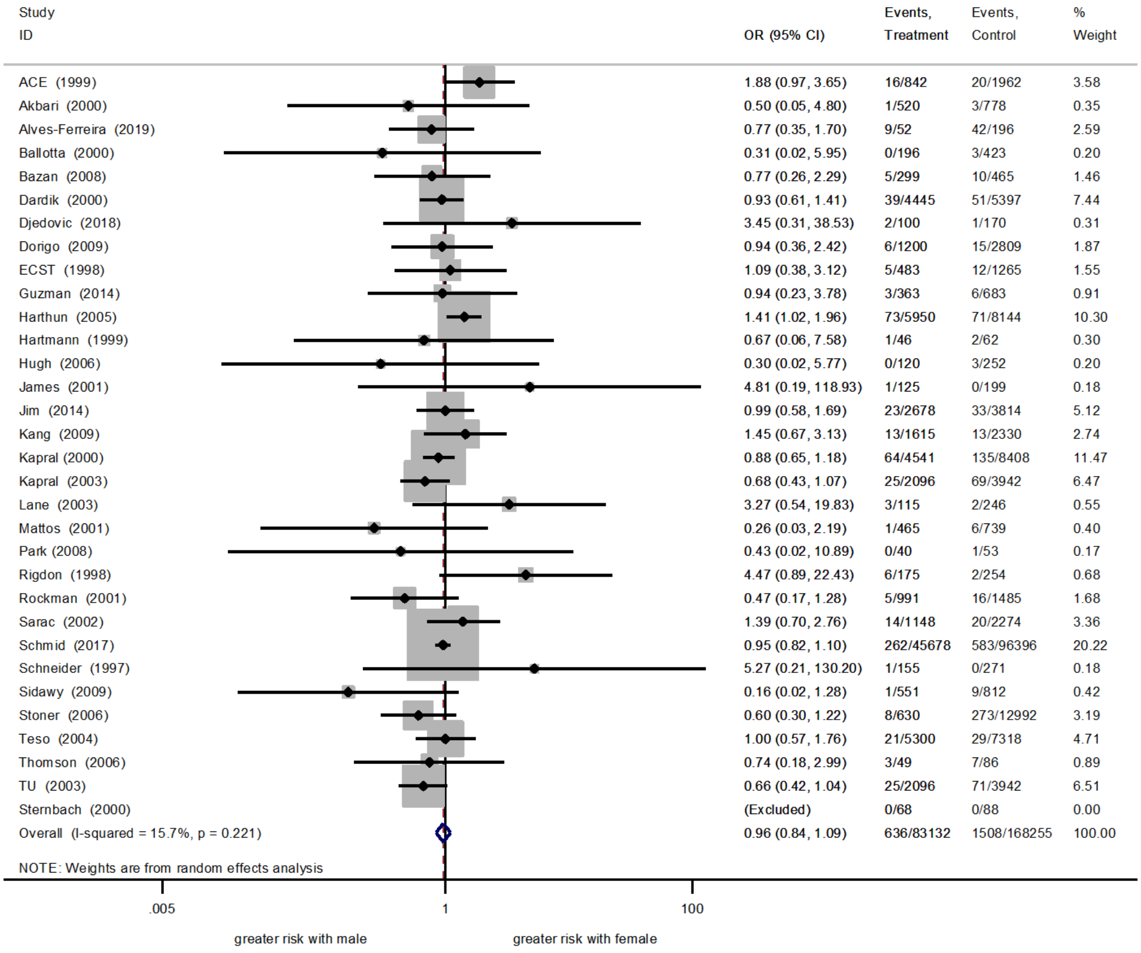

S1.7 Fig:. 5-year stroke risk between female vs male

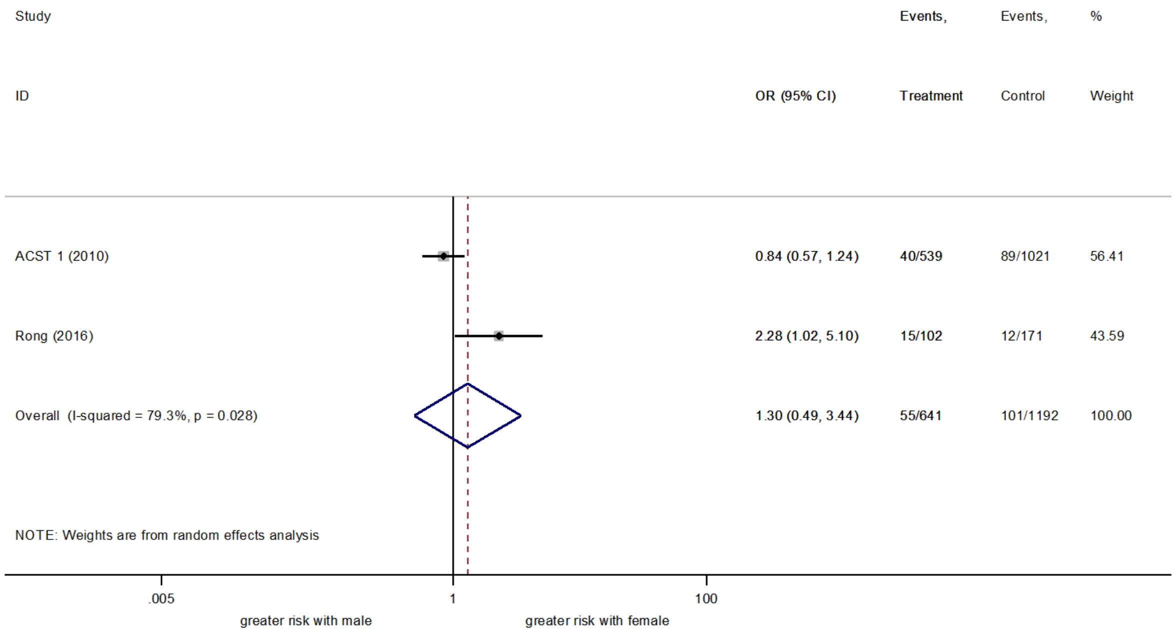

S1.8 Fig. 4-year combined stroke and death risk between female vs male

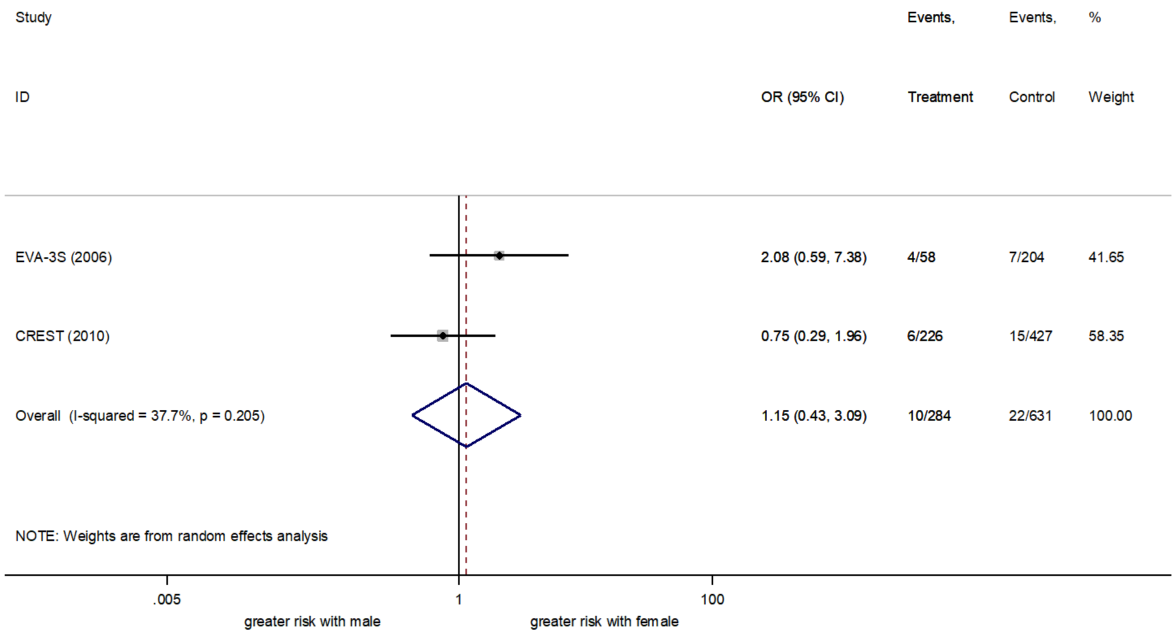

Supplement: S1 Fig — (PDF) [file pone.0285540.s002.pdf]
