# Supplementary material for: Influences of age and gender on operative risks following carotid endarterectomy: A systematic review and meta-analysis
Source: PLoS One. 2023 May 10;18(5):e0285540. doi: 10.1371/journal.pone.0285540 (PMC10171679; doi:10.1371/journal.pone.0285540)
Supplement: S2 Fig — (PDF) [file pone.0285540.s003.pdf]

S2 Fig. Publication bias

S2.1 Fig. Funnel plot (A), Egger graph (B): 30 days stroke risk between age ≥75 years vs <75 years.

(A)

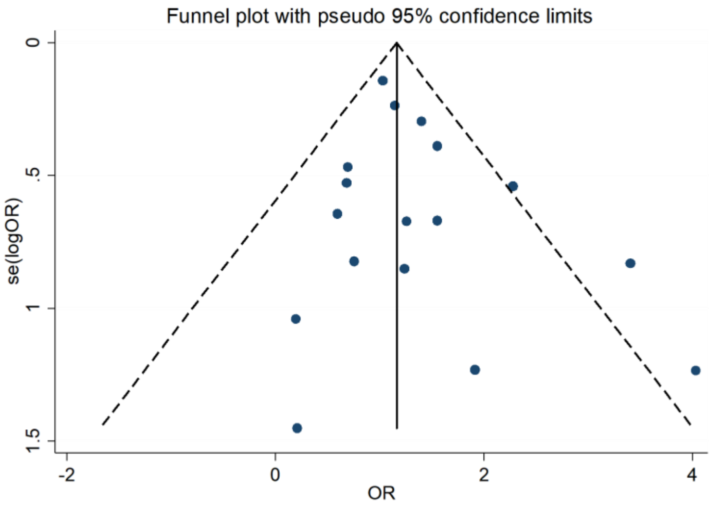

(B)

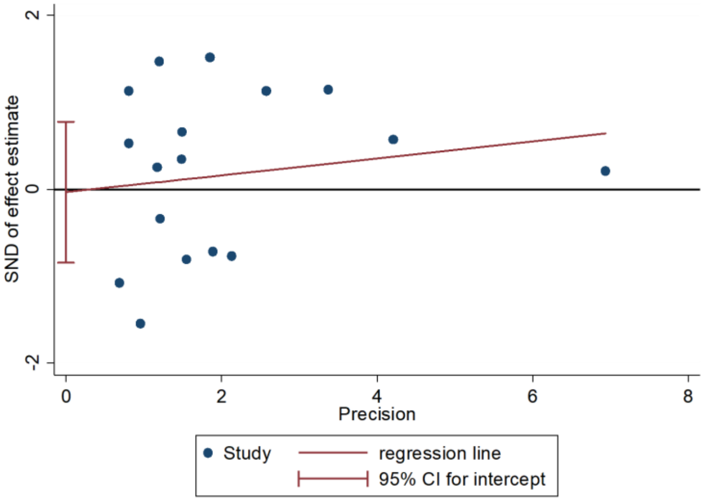

Begg's test p=0.94

Egger's test p=0.93

S2.2 Fig. Funnel plot (A), Egger graph (B), Funnel plot after performed trim-and-fill method (C): 30 days stroke risk between age ≥80 years vs <80 years.

(A)

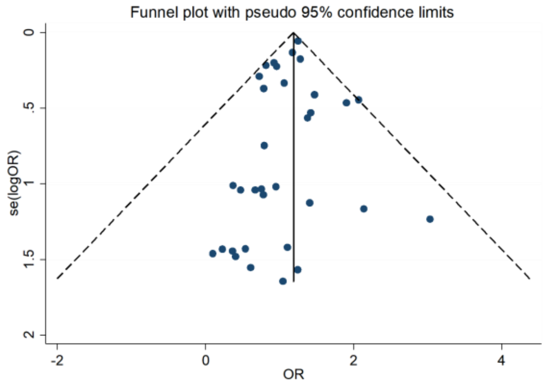

(B)

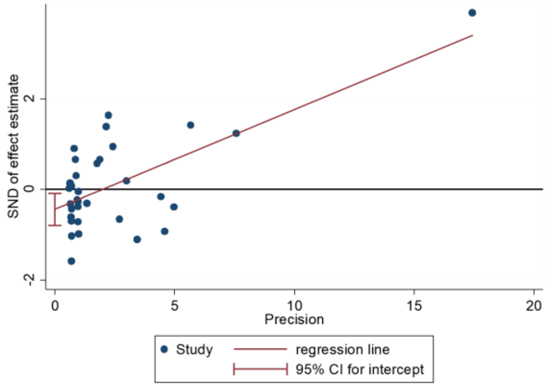

Begg’s test p=0.46
Egger’s test p=0.02

©

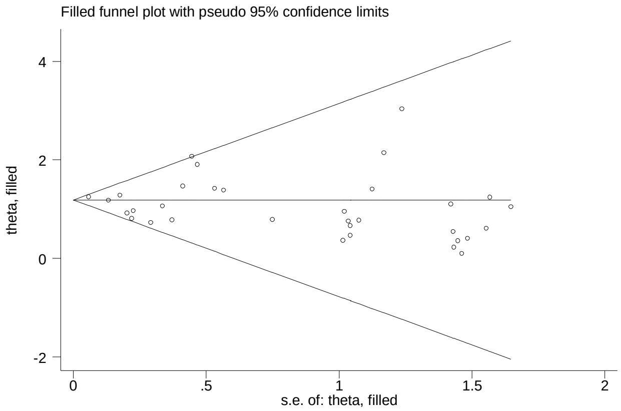

Pooled effect estimates=1.17 (2% changes)

S2.3 Fig. Funnel plot (A), Egger graph (B): 30 days death risk between age ≥75 years vs <75 years.

(A)

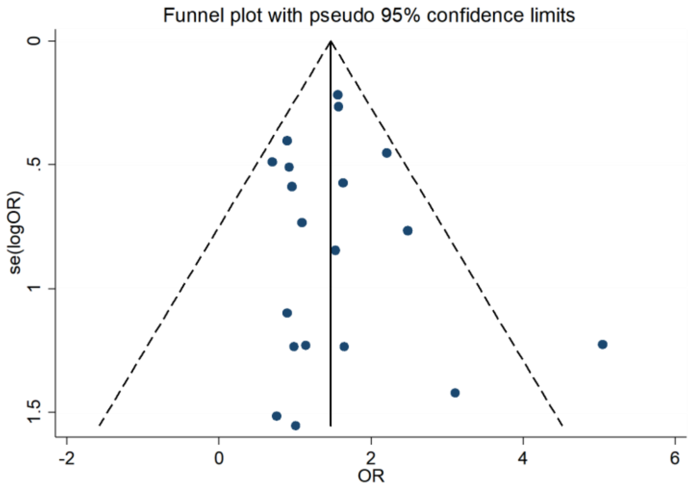

(B)

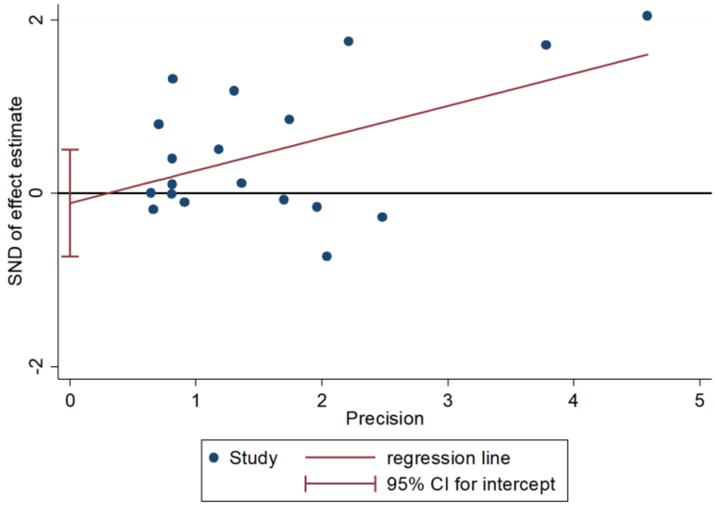

Begg's test p=0.92

Egger's test p=0.71

S2.4 Fig. Funnel plot (A), Egger graph (B), Funnel plot after performed trim-and-fill method (C): 30 days death risk between age ≥80 years vs <80 years.

(A)

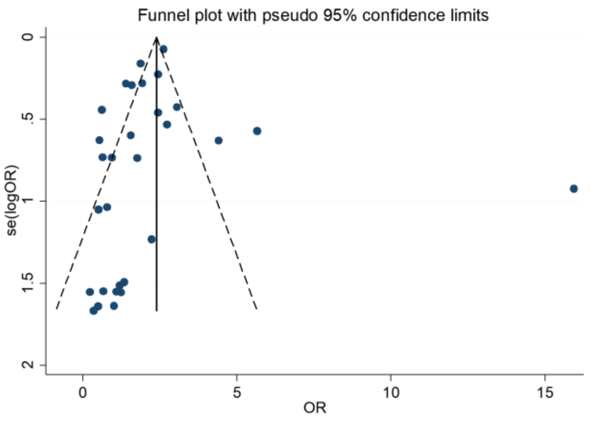

(B)

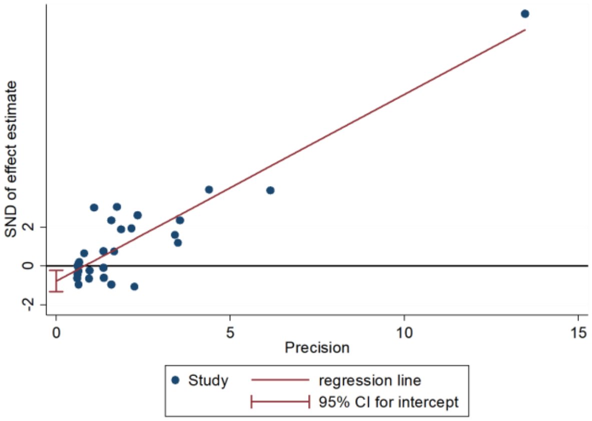

Begg's test p=0.38

Egger's test p=0.01

(C)

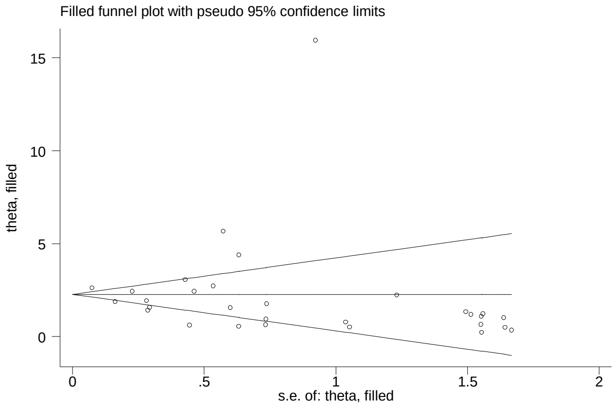

Pooled effect estimates=2.27 (23% changes)

S2.5 Fig. Funnel plot (A), Egger graph (B): 30 days combined stroke and death risk between age ≥75 years vs <75 years.

(A)

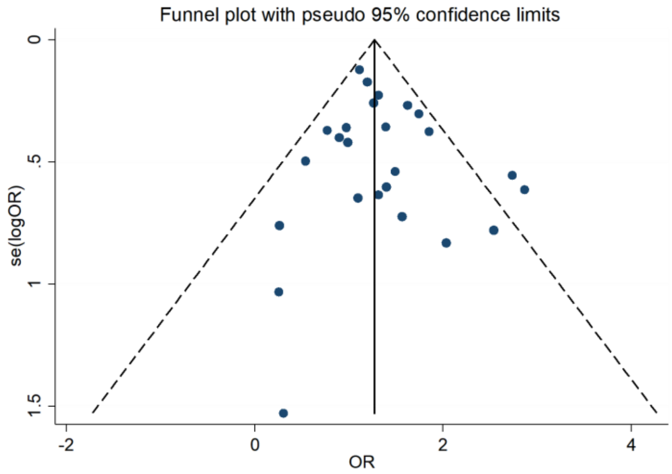

(B)

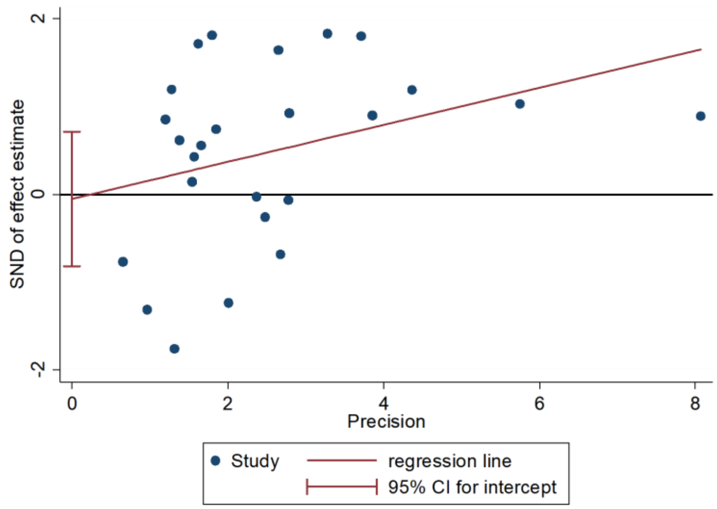

Begg's test p=0.71

Egger's test p=0.89

S2.6 Fig. Funnel plot (A), Egger graph (B), Funnel plot after performed trim-and-fill method (C): 30 days combined stroke and death risk between age ≥80 years vs <80 years.

(A)

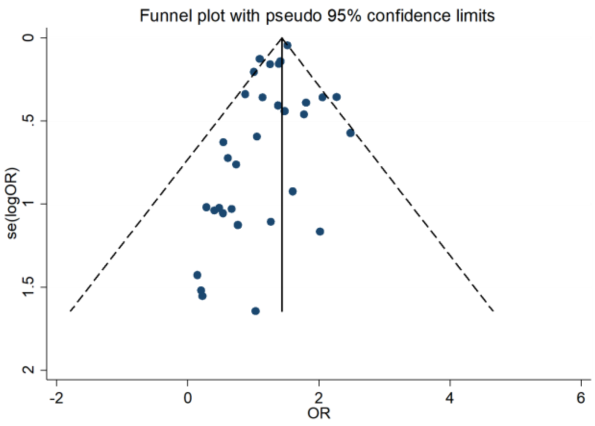

(B)

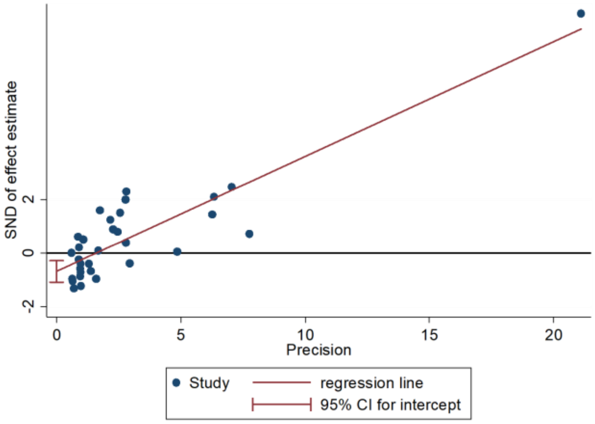

Begg's test p=0.16
Egger's test p=0.002

(C)

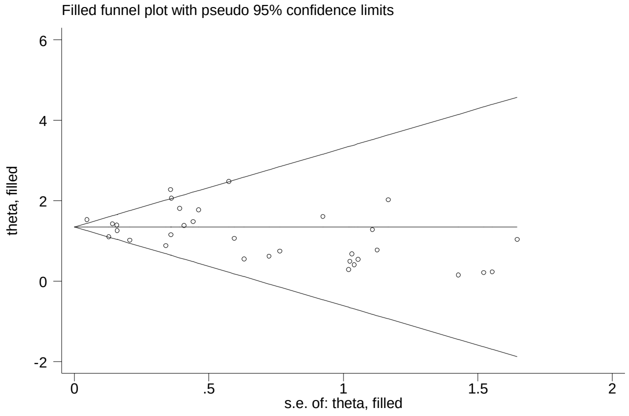

Pooled effect estimates=1.35 (2% changes)

S2.7 Fig. Funnel plot (A), Egger graph (B), Funnel plot after performed trim-and-fill method (C): 30 days stroke risk between female vs male.

(A)

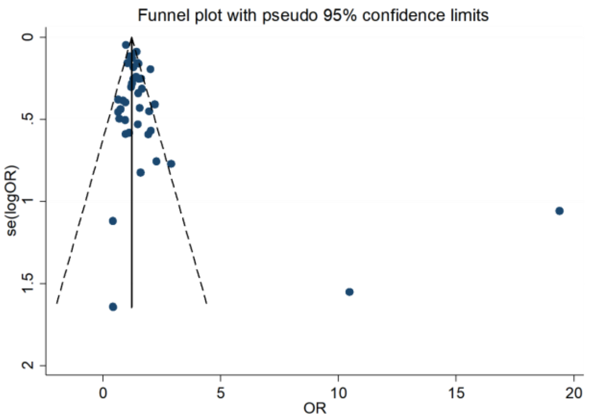

(B)

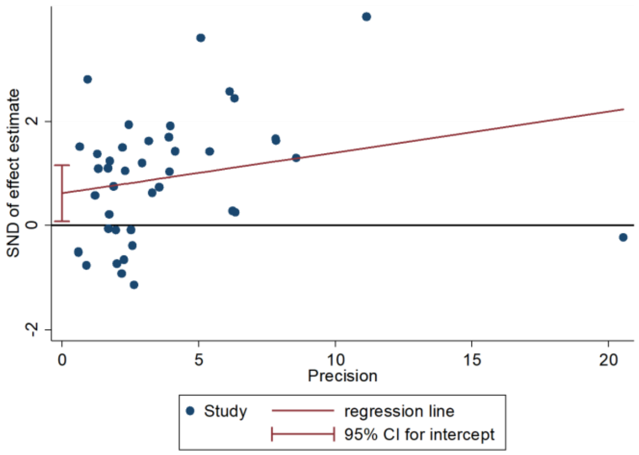

Begg's test p=0.75
Egger's test p=0.03

(C)

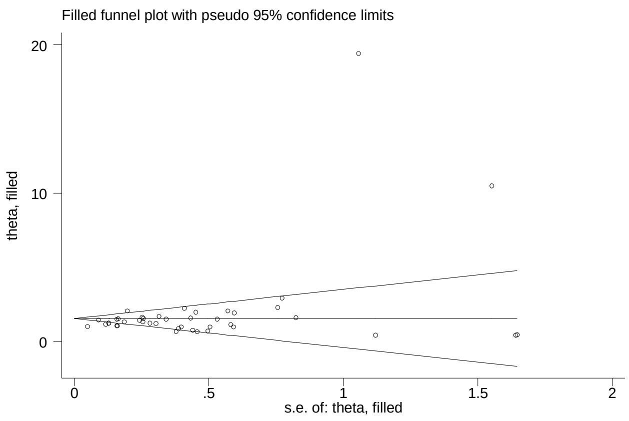

Pooled effect estimates=1.55 (21% changes)

S2.8 Fig. Funnel plot (A), Egger graph (B): 30 days death risk between female vs male.

(A)

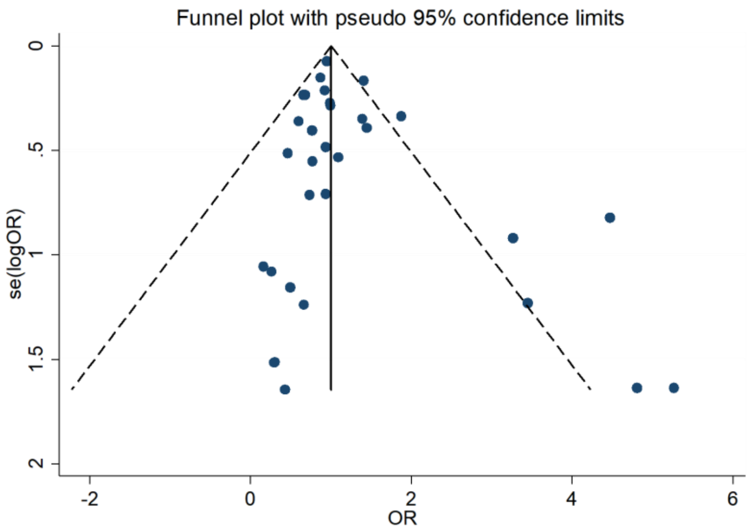

(B)

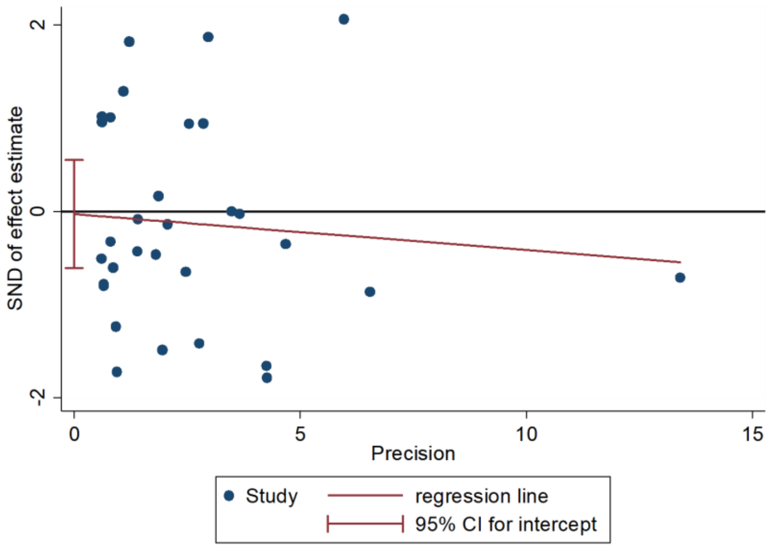

Begg's test p=0.88
Egger's test p=0.92

S2.9 Fig. Funnel plot (A), Egger graph (B): 30 days combined stroke and death risk between female vs male.

(A)

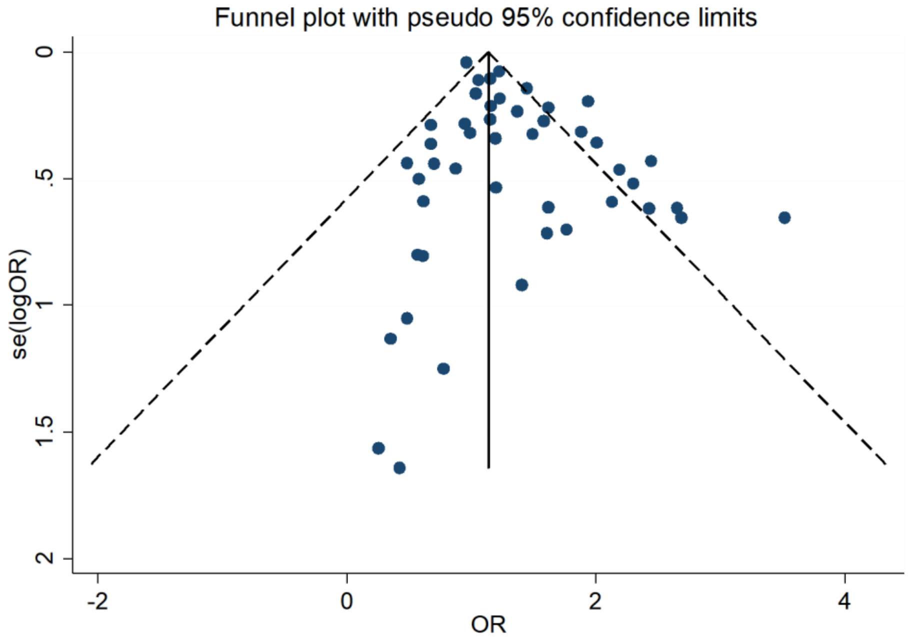

(B)

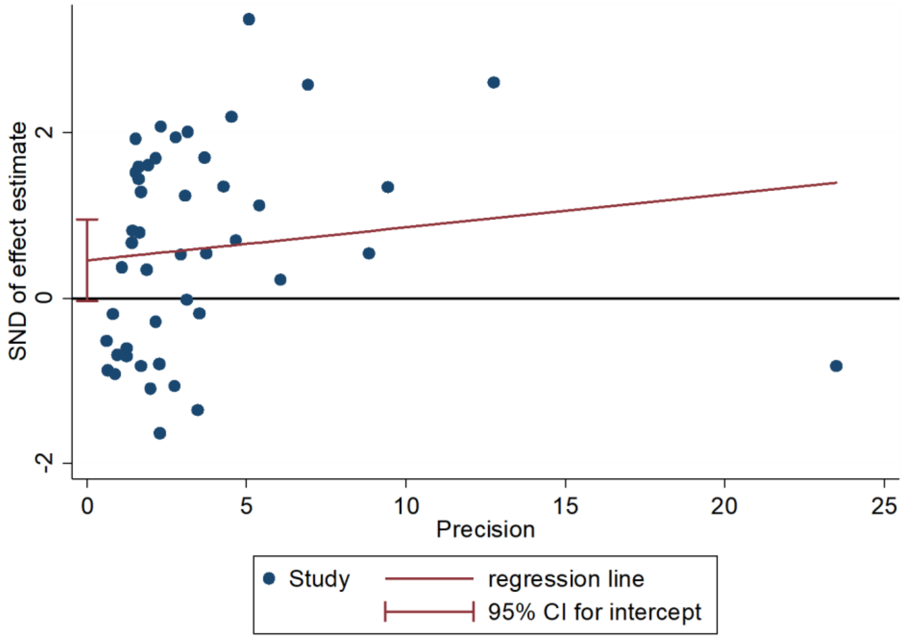

Begg’s test p=0.20
Egger’s test p=0.08
