# Supplementary material for: Influences of age and gender on operative risks following carotid endarterectomy: A systematic review and meta-analysis
Source: PLoS One. 2023 May 10;18(5):e0285540. doi: 10.1371/journal.pone.0285540 (PMC10171679; doi:10.1371/journal.pone.0285540)
Supplement: S1 Table — (PDF) [file pone.0285540.s004.pdf]

S1 Table: Search strategy

Databases	Order	Search strategy
Cochrane Library	#1	MeSH descriptor: [Endarterectomy, Carotid] explode all trees
	#2	(endarterectomy)
	#3	MeSH descriptor: [Carotid Arteries] explode all trees and with qualifier(s): [surgery-SU]
	#4	MeSH descriptor: [Carotid Arteries] explode all trees
	#5	MeSH descriptor: [Carotid Artery Diseases] explode all trees
	#6	#4 AND #5
	#7	(carotid)
	#8	(endarterectomy OR surgery)
	#9	#7 AND #8
	#10	#1 OR #2 OR #3 OR #6 OR #9
MEDLINE	1	Endarterectomy, Carotid/
	2	exp Carotid Arteries/su [Surgery]
	3	exp Carotid Artery Diseases/su [Surgery]
	4	exp carotid arteries/
	5	exp carotid artery diseases/
	6	carotid.tw.
	7	4 OR 5 OR 6
	8	endarterectomy/
	9	(endarterectom$ or surg$).tw.
	10	8 OR 9
	11	7 AND 10
	12	1 OR 2 OR 3 OR 11
	13	exp humans.sh.
	14	12 AND 13
EMBASE	1	carotid endarterectomy/
	2	carotid artery surgery/
	3	exp carotid artery disease/su
	4	exp carotid artery
	5	exp carotid artery disease/
	6	4 OR 5
	7	artery surgery/ or endarterectomy/ or vascular surgery/ or surgery/
	8	6 and 7
	9	(carotid adj5 (endarterect$ or surgery)).tw.
	10	1 OR 2 OR 3 OR 8 OR 9
