# Supplementary material for: Influences of age and gender on operative risks following carotid endarterectomy: A systematic review and meta-analysis
Source: PLoS One. 2023 May 10;18(5):e0285540. doi: 10.1371/journal.pone.0285540 (PMC10171679; doi:10.1371/journal.pone.0285540)
Supplement: S2 Table — (PDF) [file pone.0285540.s005.pdf]

S2 Table: Characteristics of the included studies

Author_Year	Recruitment period	Study design	Settings	Symptom	Age			Gender	
					Cut-off age	Sample size for analysis (Older/Younger)	Outcomes (periods)	Sample size for analysis (Female/Male)	Outcomes (periods)
ACAS_1995	March 1988-October 1993	RCT	39 clinical centers under the Asymptomatic Carotid Atherosclerosis Study Group	Asymptomatic	75	130/683	Stroke (30 days post-surgery)		
						130/695	Death (30 days post-surgery)		
						130/695	Combined stroke and death (30 days post-surgery)	281/544	Combined stroke and death (30 days post-surgery)
ACE_1999	July 1994-April 1998	RCT	74 centers: 48 in the USA, 19 in Canada, four in Australia, one in Italy, one in Argentina, and one in Finland. Most centers were participants in the North American Symptomatic Carotid Endarterectomy Trial designing study of Acetylsalicylic acid (NASCET trial)	Mixed	75	103/1642	Stroke (30 days post-surgery)	799/1962	Stroke (30 days post-surgery)
						678/2126	Death (30 days post-surgery)	842/1962	Death (30 days post-surgery)
						678/2126	Combined stroke and death (30 days post-surgery)	842/1962	Combined stroke and death (30 days post-surgery)
Ackerstaff_2000	April1990-January 1996	non-RCT	2 hospitals in the United States and the Netherlands	Mixed				287/760	Stroke (30 days post-surgery)
ACST_2004	April 1993-July 2003	RCT	The international Asymptomatic Carotid Surgery Trial (ACST), 126 hospitals in 30 countries	Asymptomatic	75	295/1110	Combined stroke and death (30 days post-surgery)		

Author_Year	Recruitment period	Study design	Settings	Symptom	Age			Gender	
					Cut-off age	Sample size for analysis (Older/Younger)	Outcomes (periods)	Sample size for analysis (Female/Male)	Outcomes (periods)
Akbari_2000	January 1990-December 1998	non-RCT	A single institution in USA	Mixed				513/778	Stroke (30 days post-surgery)
								520/778	Death (30 days post-surgery)
Alves-Ferreira_2019	2010-2018	non-RCT	2 Portuguese hospitals	Mixed				37/196	Stroke (30 days post-surgery)
								52/196	Death (30 days post-surgery)
								52/196	Stroke (5 years post-surgery)
Alozairi_2003	April 1991-April 2000	non-RCT	Aberdeen Vascular Unit, United Kingdom	Mixed	75	55/180	Stroke (30 days post-surgery)		
						55/180	Death (30 days post-surgery)		
						55/180	Combined stroke and death (30 days post-surgery)		
Ascher_2001	January 1996-December 1998	non-RCT	Division of Vascular Surgery, Department of Surgery, Maimonides Medical Center, Brooklyn, New York, USA	Symptomatic	80	109/125	Death (30 days post-surgery)		
						109/125	Combined stroke and death (30 days post-surgery)		
Aune_2003	1992-2001	non-RCT	Norway	Symptomatic	75	56/172	Stroke (30 days post-surgery)		
						56/172	Death (30 days post-surgery)		
						56/172	Combined stroke and death (30 days post-surgery)		

Author_Year	Recruitment period	Study design	Settings	Symptom	Age			Gender	
					Cut-off age	Sample size for analysis (Older/Younger)	Outcomes (periods)	Sample size for analysis (Female/Male)	Outcomes (periods)
Ballotta_2000	July 1,1989-March 31, 1998	non-RCT	The section of Vascular Surgery, University of Padua School of Medicine, Padova, Italy	Mixed				196/423	Death (30 days post-surgery)
								196/423	Combined stroke and death (30 days post-surgery)
Ballotta_2004	January 1990-December 2002	non-RCT	The section of Vascular Surgery, University of Padua School of Medicine, Padova, Italy	Mixed	80	54/81	Death (30 days post-surgery)		
						54/81	Combined stroke and death (30 days post-surgery)		
Bazan_2008	1992-2002	non-RCT	The Connecticut Hospital Association (CHA) Data Program, USA	Symptomatic	80	153/611	Stroke (30 days post-surgery)	299/465	Stroke (30 days post-surgery)
						153/611	Death (30 days post-surgery)	299/465	Death (30 days post-surgery)
						153/611	Combined stroke and death (30 days post-surgery)	299/465	Combined stroke and death (30 days post-surgery)
Blohme_1999	1990-1994	non-RCT	The Department of Surgery, Karolinska Hospital, Sweden	Symptomatic				94/178	Combined stroke and death (30 days post-surgery)
Brott_2010 (CREST)	December 2000-July 2008	RCT	CREST trial	Mixed	70	326/327	Combined stroke and death (4 years post-surgery)		
Brown_2008	1997-2006	non-RCT	Veterans Affairs Medical Center, Chicago, Illinois, USA	Mixed	80	82/9	Stroke (30 days post-surgery)		
						82/9	Death (30 days post-surgery)		

Author_Year	Recruitment period	Study design	Settings	Symptom	Age			Gender	
					Cut-off age	Sample size for analysis (Older/Younger)	Outcomes (periods)	Sample size for analysis (Female/Male)	Outcomes (periods)
Calvillo-king_2010	January 1998-June 1999	non-RCT	Medicare beneficiaries in New York State, USA	Asymptomatic	80	1549/5104	Stroke (30 days post-surgery)	3011/3655	Stroke (30 days post-surgery)
						1549/5104	Combined stroke and death (30 days post-surgery)	2996/3655	Combined stroke and death (30 days post-surgery)
Cartier_2002	July 1990-April 2001	non-RCT	Suroit Regional Hospital, Salaberry-de-Valleyfield, Canada	Mixed	80	72/403	Stroke (30 days post-surgery)		
						72/403	Death (30 days post-surgery)		
						72/403	Combined stroke and death (30 days post-surgery)		
Cebul_1998	July 1993-June 1994	non-RCT	Ohio hospitals, USA	Mixed	80	110/568	Combined stroke and death (30 days post-surgery)		
Coyle_1994	January 1983-December 1992	non-RCT	Emory University Hospital, Atlanta, Georgia	Mixed	80	79/992	Stroke (30 days post-surgery)		
						79/992	Death (30 days post-surgery)		
						79/992	Combined stroke and death (30 days post-surgery)		
Dardik_2000	1990-1995	non-RCT	All nonfederal acute care hospitals in the state	Mixed				4432/5397	Stroke (30 days post-surgery)
								4445/5397	Death (30 days post-surgery)
De Aguiar_2001	January 1987-December 1997	non-RCT	A tertiary care private hospital in São Paulo, Brazil	Mixed				26/41	Combined stroke and death (30 days post-surgery)

Author_Year	Recruitment period	Study design	Settings	Symptom	Age			Gender	
					Cut-off age	Sample size for analysis (Older/Younger)	Outcomes (periods)	Sample size for analysis (Female/Male)	Outcomes (periods)
Debing_2007	March 1988-November 2005	non-RCT	Academic Hospital, Free University of Brussels, Brussels, Belgium	Mixed	75	286/742	Stroke (30 days post-surgery)		
						286/742	Death (30 days post-surgery)		
						286/742	Combined stroke and death (30 days post-surgery)		
De Waard_2017	-	RCT	The ACST-1	Asymptomatic				476/945	Stroke (30 days post-surgery)
Djedovic_2018	January 2012-October 2017	non-RCT	The Clinical Center of the University of Sarajevo	Mixed				101/170	Stroke (30 days post-surgery)
								100/170	Death (30 days post-surgery)
								100/170	Combined stroke and death (30 days post-surgery)
Doonan_2019	October 2009-Dcember 2015	non-RCT	The Jewish General Hospital, Montreal, Canada and Royal Victoria Hospital, UK	Mixed	80	65/296	Stroke (30 days post-surgery)		
						65/296	Death (30 days post-surgery)		
						65/296	Stroke (5 years post-surgery)		
						65/296	Death (5 years post-surgery)		
Dorafshar_2004	January 2000-December 2001	non-RCT	UCLA Medical Center, Los Angeles, USA	Mixed	80	30/96	Stroke (30 days post-surgery)		
						30/95	Death (30 days post-surgery)		
						30/95	Combined stroke and death (30 days post-surgery)		

Author_Year	Recruitment period	Study design	Settings	Symptom	Age			Gender	
					Cut-off age	Sample size for analysis (Older/Younger)	Outcomes (periods)	Sample size for analysis (Female/Male)	Outcomes (periods)
Dorigo_2009	January 1996-December 2007	non-RCT	University of Florence, Italy	Mixed	80	190/1010	Combined stroke and death (30 days post-surgery)	1187/2809	Stroke (30 days post-surgery)
								1200/2809	Death (30 days post-surgery)
								1200/2309	Combined stroke and death (30 days post-surgery)
Dulai_2019	July 2003-May 2016	non-RCT	The tertiary vascular center in Galway, Ireland	Mixed				209/432	Stroke (30 days post-surgery)
Eckstein_2002	March 1997-August 2000	non-RCT	The multicenter in Germany	Symptomatic				45/119	Combined stroke and death (30 days post-surgery)
ECST_1998	October 1981-March 1994	RCT	97 centers in 12 European countries and 1 center in Australia	Symptomatic	75	103/1642	Stroke (30 days post-surgery)	463/1262	Stroke (30 days post-surgery)
						103/1642	Death (30 days post-surgery)	483/1265	Death (30 days post-surgery)
						103/1642	Combined stroke and death (30 days post-surgery)	483/1262	Combined stroke and death (30 days post-surgery)
Ederle_2009	March 1992-July 1997	RCT	The 22 trial centers in western Europe, Australia, or Canada	Mixed				75/178	Combined stroke and death (30 days post-surgery)
Fisher_1989	-	non-RCT	-	Mixed	80	685/1404	Death (30 days post-surgery)		
Frawley_2000	July 1985-October 1997	non-RCT	-	Mixed				309/688	Stroke (30 days post-surgery)

Author_Year	Recruitment period	Study design	Settings	Symptom	Age			Gender	
					Cut-off age	Sample size for analysis (Older/Younger)	Outcomes (periods)	Sample size for analysis (Female/Male)	Outcomes (periods)
Friedmann_1988	1971-1988	non-RCT	A community-based teaching hospital in Boston, Massachusetts	Mixed				272/408	Stroke (30 days post-surgery)
Glousman_2020	2012-2017	non-RCT	The American College of Surgeons National Surgical Quality Improvement Program (ACS-NSQIP) database	Asymptomatic	80	2509/11337	Stroke (30 days post-surgery)		
						2509/11337	Death (30 days post-surgery)		
						2509/11337	Combined stroke and death (30 days post-surgery)		
Goldman_1999	April 1995-May 1998	non-RCT	A single 224-bed community hospital, New Jersey, USA	Mixed	80	76/234	Stroke (30 days post-surgery)		
						76/234	Death (30 days post-surgery)		
						76/234	Combined stroke and death (30 days post-surgery)		
Goldstein_1994	1988-1990	non-RCT	11 hospitals of Academic Medical Center Consortium, New York, USA	Mixed	75	137/560	Combined stroke and death (30 days post-surgery)	256/441	Combined stroke and death (30 days post-surgery)
Goodney_2008	January 2003-December 2007	non-RCT	11 hospitals in Northern New England	Mixed				1268/1824	Combined stroke and death (30 days post-surgery)

Author_Year	Recruitment period	Study design	Settings	Symptom	Age			Gender	
					Cut-off age	Sample size for analysis (Older/Younger)	Outcomes (periods)	Sample size for analysis (Female/Male)	Outcomes (periods)
Grego_2005	January 1996-December 2002	non-RCT	Padua University Hospital, Italy	Mixed	80	132/1601	Stroke (30 days post-surgery)		
						132/1601	Death (30 days post-surgery)		
						132/1601	Combined stroke and death (30 days post-surgery)		
Guzmn_2014	January 1, 1993-December 15, 2010	non-RCT	-	Mixed				357/683	Stroke (30 days post-surgery)
								363/683	Death (30 days post-surgery)
								363/683	Combined stroke and death (30 days post-surgery)
Halm_2005	January 1997-December 1998	non-RCT	6 Hospitals, New York, USA	Mixed	80	414/1558	Stroke (30 days post-surgery)	848/1122	Stroke (30 days post-surgery)
						414/1558	Combined stroke and death (30 days post-surgery)	850/1122	Combined stroke and death (30 days post-surgery)
Halm_2009	January 1998-June 1999	non-RCT	The New York State hospital discharge database, New York, USA	Mixed	80	2197/7110	Stroke (30 days post-surgery)	4112/5181	Stroke (30 days post-surgery)
						7156/2152	Combined stroke and death (30 days post-surgery)	4125/5181	Combined stroke and death (30 days post-surgery)
Halliday_2010 (ACST-1)	1993-2003	RCT	The ACST-1	Asymptomatic				539/1021	Stroke (5 years post-surgery)
Harthun_2005	1997-2001	non-RCT	Non-Federal acute-care Virginia hospitals	Mixed				5951/8144	Stroke (30 days post-surgery)
								5950/8144	Death (30 days post-surgery)

Author_Year	Recruitment period	Study design	Settings	Symptom	Age			Gender	
					Cut-off age	Sample size for analysis (Older/Younger)	Outcomes (periods)	Sample size for analysis (Female/Male)	Outcomes (periods)
Hartmann_1999	April 1995-March 1997	non-RCT	The Universität KLINIKUM Benjamin Franklin of the Freie Universität Berlin	Mixed				48/62	Stroke (30 days post-surgery)
								46/62	Death (30 days post-surgery)
								46/62	Combined stroke and death (30 days post-surgery)
Hartzer_1997	January 1989-1995	non-RCT	The Department of Vascular Surgery, and the Department of Biostatistics and Epidemiology, Cleveland Clinic Foundation in USA	Mixed				652/1272	Combined stroke and death (30 days post-surgery)
Hoffmann_2008 (BACASS)	November 1998-February 2002	RCT	BACASS trial	Symptomatic	70	1/9	Combined stroke and death (2 years post-surgery)	1/9	Combined stroke and death (2 years post-surgery)
Hugh_2006	1997-2003	non-RCT	-	Mixed				122/252	Stroke (30 days post-surgery)
								120/252	Death (30 days post-surgery)
								120/252	Combined stroke and death (30 days post-surgery)
James_2001	January 1995-December 1999	non-RCT	University of Arizona Health Sciences Center	Mixed				125/199	Stroke (30 days post-surgery)
								125/199	Death (30 days post-surgery)
								125/199	Combined stroke and death (30 days post-surgery)

Author_Year	Recruitment period	Study design	Settings	Symptom	Age			Gender	
					Cut-off age	Sample size for analysis (Older/Younger)	Outcomes (periods)	Sample size for analysis (Female/Male)	Outcomes (periods)
Jeong_2019	January 2007-December 2014	non-RCT	University of Ulsan College of Medicine and Asan Medical Center, Seoul, Republic of Korea	Mixed	60	572/103	Stroke (30 days post-surgery)		
						572/103	Death (30 days post-surgery)		
						572/103	Stroke (5 years post-surgery)		
						572/103	Death (5 years post-surgery)		
Jim_2012	July 2005-December 2010	non-RCT	The Society for Vascular Surgery Vascular Registry database (SVS-VR), USA	Mixed	65	4169/1347	Stroke (30 days post-surgery)		
						4169/1347	Death (30 days post-surgery)		
Jim_2014	-	non-RCT	The Society for Vascular Surgery Vascular Registry database (SVS-VR), USA	Mixed				2652/3814	Stroke (30 days post-surgery)
								2678/3814	Death (30 days post-surgery)
Jordan_2002	January 1, 1998-December 31, 2000	non-RCT	The University of Alabama at Birmingham and the Birmingham Veteran’s Affairs Medical Center, UK	Mixed				127/288	Combined stroke and death (30 days post-surgery)
Kang_2009	January 2005-December 2006	non-RCT	The American College of Surgeons National Surgical Quality Improvement Program (ACS-NSQIP) database	Mixed	80	3212/737	Stroke (30 days post-surgery)	1621/2330	Stroke (30 days post-surgery)
						3212/737	Death (30 days post-surgery)	1615/2330	Death (30 days post-surgery)

Author_Year	Recruitment period	Study design	Settings	Symptom	Age			Gender	
					Cut-off age	Sample size for analysis (Older/Younger)	Outcomes (periods)	Sample size for analysis (Female/Male)	Outcomes (periods)
Kapral_2000	1982-1994	non-RCT	The Canadian Institute for Health Information (CIHI)	Mixed				4465/8408	Stroke (30 days post-surgery)
								4541/8408	Death (30 days post-surgery)
								4541/8408	Combined stroke and death (30 days post-surgery)
Kapral_2003	1994-1997	non-RCT	The Ontario Carotid Endarterectomy Registry	Mixed				2034/3942	Stroke (30 days post-surgery)
								2096/3942	Death (30 days post-surgery)
								2096/3942	Combined stroke and death (30 days post-surgery)
Karp_1998	1993	non-RCT	Georgia, USA	Mixed	75	700/1245	Combined stroke and death (30 days post-surgery)	910/1035	Combined stroke and death (30 days post-surgery)
Kazmers_1999	1991-1994	non-RCT	Veterans Affairs Medical Centers	Mixed	80	195/8957	Death (30 days post-surgery)		
Kerdiles_1997	June 1985-December 1993	non-RCT	CHU Rennes - Hôpital Sud - Maternité, Rennes, France	Mixed	75	281/741	Stroke (30 days post-surgery)		
						281/741	Death (30 days post-surgery)		
						281/741	Combined stroke and death (30 days post-surgery)	103/178	Combined stroke and death (30 days post-surgery)
Khatri_2012	2005-2008	non-RCT	The Nationwide Inpatient Sample (NIS) database, USA	Mixed	70	256826/180827	Stroke (30 days post-surgery)		
						256826/180827	Death (30 days post-surgery)		

Author_Year	Recruitment period	Study design	Settings	Symptom	Age			Gender	
					Cut-off age	Sample size for analysis (Older/Younger)	Outcomes (periods)	Sample size for analysis (Female/Male)	Outcomes (periods)
Knappich_2019	-	RCT	5 trials combined (EVA-3S (NCT00190398, 527 patients), SPACE (ISRCTN57874028, 1214 patients), ICSS (ISRCTN25337470, 1713 patients), and CREST (NCT00004732, 2502 patients in total of whom 1321 had a symptomatic carotid stenosis)	Symptomatic				1234/2971	Combined stroke and death (30 days post-surgery)
Kucey_1998	January 1994-December 1996	non-RCT	8 University of Toronto–affiliated hospitals, Canada	Mixed	75	295/983	Stroke (30 days post-surgery)		
						295/983	Death (30 days post-surgery)		
						295/983	Combined stroke and death (30 days post-surgery)	434/847	Combined stroke and death (30 days post-surgery)
Lane_2003	1988-1998	non-RCT	University of California-San Francisco and University of California-Los Angeles, USA	Mixed				116/246	Stroke (30 days post-surgery)
								115/246	Death (30 days post-surgery)
								115/246	Combined stroke and death (30 days post-surgery)

Author_Year	Recruitment period	Study design	Settings	Symptom	Age			Gender	
					Cut-off age	Sample size for analysis (Older/Younger)	Outcomes (periods)	Sample size for analysis (Female/Male)	Outcomes (periods)
Lau_2005	January 1995-December 2004	non-RCT	The John D. Dingell Veterans Administration (VA) medical center database, Detroit, Michigan, USA	Mixed	80	39/247	Stroke (30 days post-surgery)		
						39/247	Death (30 days post-surgery)		
						39/247	Combined stroke and death (30 days post-surgery)		
Love_2000	February 1979-February 1985 January 1995-December 1998	non-RCT	Greenslopes Private Hospital, Brisbane, Australia	Mixed	75	135/308	Stroke (30 days post-surgery)		
						135/308	Death (30 days post-surgery)		
						135/308	Combined stroke and death (30 days post-surgery)		
Lubke_2015	January 2000-December 2010	non-RCT	A single university vascular surgical center in Cologne, Germany	Mixed				574/1296	Stroke (30 days post-surgery)
Magnadottir_1999	November 1992-October 1998	non-RCT	N/A	Mixed	75	208/392	Combined stroke and death (30 days post-surgery)		
Magnan_1993	Series I; January 1, 1980-December 31, 1982 and series II; January 1, 1990-December 31, 1991	non-RCT	-	Mixed				90/300	Combined stroke and death (30 days post-surgery)

Author_Year	Recruitment period	Study design	Settings	Symptom	Age			Gender	
					Cut-off age	Sample size for analysis (Older/Younger)	Outcomes (periods)	Sample size for analysis (Female/Male)	Outcomes (periods)
Mas_2006 (EVA-3S)	2003-2005	RCT	EVA-3S trial	Symptomatic	70	156/109	Combined stroke and death (4 years post-surgery)		
Mattos_2001	March 1976-October 1997	non-RCT	Southern Illinois University School of Medicine, UK	Mixed				450/739	Stroke (30 days post-surgery)
								465/739	Death (30 days post-surgery)
Maxwell_1990	January 1979-December 1988	non-RCT	New Hanover Memorial Hospital, North Carolina, USA	Mixed	75	170/640	Stroke (30 days post-surgery)		
						170/640	Death (30 days post-surgery)		
						170/640	Combined stroke and death (30 days post-surgery)	289/345	Combined stroke and death (30 days post-surgery)
Maxwell_2000	1979-1998	non-RCT	New Hanover Regional Medical Center, North Carolina, USA	Mixed	80	218/2180	Stroke (30 days post-surgery)		
						218/2180	Death (30 days post-surgery)		
						218/2180	Combined stroke and death (30 days post-surgery)		
Mazzalai_2009	1990-2007	non-RCT	N/A	Mixed	80	159/1341	Stroke (30 days post-surgery)		
						159/1341	Death (30 days post-surgery)		
Middleton_2002	1983-1997	non-RCT	New South Wales, Australia	Mixed	75	228/414	Death (30 days post-surgery)		

Author_Year	Recruitment period	Study design	Settings	Symptom	Age			Gender	
					Cut-off age	Sample size for analysis (Older/Younger)	Outcomes (periods)	Sample size for analysis (Female/Male)	Outcomes (periods)
Miller_2005	1993-2004	non-RCT	The Jobst Vascular Registry at Toledo Hospital, Ohio, USA	Mixed	80	360/1857	Stroke (30 days post-surgery)		
						360/1857	Death (30 days post-surgery)		
						360/1857	Combined stroke and death (30 days post-surgery)		
NASCET_1991	January 1988-February 1991	RCT	50 clinical centers in US and Canada (NASCET trial)	Symptomatic				250/509	Stroke (2 years post-surgery)
Navas_2008	2000-2006	non-RCT	N/A	Symptomatic	75	26/60	Combined stroke and death (30 days post-surgery)		
Naylor_2000	October 1995-January 1999	non-RCT	Leicestershire, England	Mixed	75	115/385	Combined stroke and death (30 days post-surgery)	171/329	Combined stroke and death (30 days post-surgery)
Nunnelee_1995	January 1979-December 1991	non-RCT	N/A	Mixed	75	532/952	Stroke (30 days post-surgery)		
						232/952	Death (30 days post-surgery)		
						232/952	Combined stroke and death (30 days post-surgery)		

Author_Year	Recruitment period	Study design	Settings	Symptom	Age			Gender	
					Cut-off age	Sample size for analysis (Older/Younger)	Outcomes (periods)	Sample size for analysis (Female/Male)	Outcomes (periods)
Okawa_2015	May 2008-April 2013	non-RCT	Fukuoka University Hospital, Japan	Mixed	80	19/123	Stroke (30 days post-surgery)		
						19/123	Death (30 days post-surgery)		
						19/120	Stroke (5 years post-surgery)		
						19/120	Death (5 years post-surgery)		
Ommer_2001	1990-1999	non-RCT	Department for Vascular Surgery and Kidney Transplantation at the Heinrich-Heine-University, Duesseldorf, Germany	Mixed	80	76/2186	Stroke (30 days post-surgery)		
						76/2186	Death (30 days post-surgery)		
						76/2186	Combined stroke and death (30 days post-surgery)		
Organ_2008	1992-2007	non-RCT	Royal Brisbane and Women's Hospital, Herston, Queensland, Australia	Mixed	80	118/1195	Combined stroke and death (30 days post-surgery)	407/909	Combined stroke and death (30 days post-surgery)
Ouriel_1986	N/A	non-RCT	N/A	Mixed	75	77/393	Stroke (30 days post-surgery)		
						77/393	Death (30 days post-surgery)		
						77/393	Combined stroke and death (30 days post-surgery)		

Author_Year	Recruitment period	Study design	Settings	Symptom	Age			Gender	
					Cut-off age	Sample size for analysis (Older/Younger)	Outcomes (periods)	Sample size for analysis (Female/Male)	Outcomes (periods)
Ozsvath_2002	January 1990-December 2000	non-RCT	Institute for Vascular Health and Disease, Albany Medical College, New York, USA	Mixed	80	125/3927	Stroke (30 days post-surgery)		
Park_2008	November 2003-December 2006	non-RCT	University of Connecticut, USA	Mixed				39/53	Stroke (30 days post-surgery)
								40/53	Death (30 days post-surgery)
								40/53	Combined stroke and death (30 days post-surgery)
Pasin_2019	June 2009-December 2014	non-RCT	N/A	N/A	80	439/2024	Combined stroke and death (5 years post-surgery)		
Papachristou_1994	1982-1988	non-RCT	The Heidehaus Municipal Hospital, Hannover, Germany	Mixed	75	84/320	Stroke (30 days post-surgery)		
						84/320	Death (30 days post-surgery)		
						84/320	Combined stroke and death (30 days post-surgery)		
Perler_1996	January 1990-December 1995	non-RCT	N/A	Mixed	75	63/124	Stroke (30 days post-surgery)		
						58/115	Death (30 days post-surgery)		
						58/115	Combined stroke and death (30 days post-surgery)		

Author_Year	Recruitment period	Study design	Settings	Symptom	Age			Gender	
					Cut-off age	Sample size for analysis (Older/Younger)	Outcomes (periods)	Sample size for analysis (Female/Male)	Outcomes (periods)
Perler_1998	1993-1994	non-RCT	The Maryland Health Services Cost Review Commission (MHSCRC) database at Johns Hopkins, Maryland, USA	Mixed	80	1036/8882	Stroke (30 days post-surgery)		
						1036/8882	Death (30 days post-surgery)		
						1036/8882	Combined stroke and death (30 days post-surgery)		
Pinkerton_1990	March 1974-July 1989	non-RCT	St. Luke's Hospital of Kansas City, Missouri, USA	Mixed	75	125/560	Stroke (30 days post-surgery)		
						125/560	Death (30 days post-surgery)		
						125/560	Combined stroke and death (30 days post-surgery)		
Pinkerton_2002	1974-2001	non-RCT	Saint Luke’s Hospital of Kansas City, USA	Mixed				613/1045	Stroke (30 days post-surgery)
Plecha_1985	1973-1983	non-RCT	The Cleveland Vascular Society registry, Ohio, USA	Mixed	75	782/5220	Death (30 days post-surgery		
Plestis_1996	January 1981-December 1993	non-RCT	-	Mixed				396/610	Combined stroke and death (30 days post-surgery)
Pol_2013	January 2005-July 2010	non-RCT	1 Academic center and 1 large community hospital, Netherlands	Symptomatic	80	71/477	Stroke (30 days post-surgery)		
						71/477	Stroke (5 years post-surgery)		

Author_Year	Recruitment period	Study design	Settings	Symptom	Age			Gender	
					Cut-off age	Sample size for analysis (Older/Younger)	Outcomes (periods)	Sample size for analysis (Female/Male)	Outcomes (periods)
Pruner_2003	January 1995-December 2000	non-RCT	Department of Vascular Surgery of the IRCCS San Raffaele Hospital of Milan, Italy	Mixed	80	245/3085	Stroke (30 days post-surgery)		
						345/3085	Death (30 days post-surgery		
						345/3085	Combined stroke and death (30 days post-surgery)		
Pulli_2005	1996-2001	non-RCT	Department of Vascular Surgery, University of Florence, Italy	Mixed	80	149/1734	Combined stroke and death (30 days post-surgery)	566/1317	Combined stroke and death (30 days post-surgery)
Rajamani_2013	January 2005-March 2011	non-RCT	The Carotid Artery Revascularization and Endarterectomy (CARE) registry, USA	Mixed	75	2760/1389	Stroke (30 days post-surgery)		
						2760/1389	Combined stroke and death (30 days post-surgery)		
Rantner_2006	-	non-RCT	-	Symptomatic				49/166	Stroke (30 days post-surgery)
Reed_2003	January 1990-December 1999	non-RCT	Brigham and Women's Hospital, Massachusetts, USA	Mixed	80	160/1210	Stroke (30 days post-surgery)		
						160/1210	Death (30 days post-surgery		
						160/1210	Combined stroke and death (30 days post-surgery)		
Rigdon_1998	-	non-RCT	-	Mixed				178/254	Stroke (30 days post-surgery)
								175/254	Death (30 days post-surgery)

Author_Year	Recruitment period	Study design	Settings	Symptom	Age			Gender	
					Cut-off age	Sample size for analysis (Older/Younger)	Outcomes (periods)	Sample size for analysis (Female/Male)	Outcomes (periods)
Riles_1994	1965-1991	non-RCT	The Division of Vascular Surgery of New York University Medical Center, USA	Mixed				799/1488	Stroke (30 days post-surgery)
Rockman_2001	1982-1997	non-RCT	The New York University Medical Center, USA	Mixed				986/1485	Stroke (30 days post-surgery)
								991/1485	Death (30 days post-surgery)
Rockman_2003	1997-1999	non-RCT	The Division of Vascular Surgery at the New York University Medical Center database, New York, USA	Mixed	80	160/535	Death (30 days post-surgery		
						160/535	Combined stroke and death (30 days post-surgery)		
Rong_2016	January 1990-December 2013	non-RCT	The neurosurgical department of Johns Hopkins Medical Institutions, USA	Mixed				105/171	Stroke (30 days post-surgery)
								102/171	Stroke (5 years post-surgery)
Salameh_2002	January 1994-December 1998	non-RCT	Department of Surgery, Inova Fairfax Hospital, Fairfax, Virginia, USA	Mixed	80	42/251	Stroke (30 days post-surgery)		
						161/537	Death (30 days post-surgery		
						42/251	Combined stroke and death (30 days post-surgery)		
Salomon_2014	January 2002 and December 2007	non-RCT	Vascular Surgery Unit, University Hospital of Besancon, Besancon, France	Mixed				48/70	Combined stroke and death (30 days post-surgery)

Author_Year	Recruitment period	Study design	Settings	Symptom	Age			Gender	
					Cut-off age	Sample size for analysis (Older/Younger)	Outcomes (periods)	Sample size for analysis (Female/Male)	Outcomes (periods)
Sarac_2002	January 1989-November 2000	non-RCT	The Department of Vascular Surgery, The Cleveland Clinic Foundation, USA	Mixed				1141/2274	Stroke (30 days post-surgery)
								1148/2274	Death (30 days post-surgery)
								1148/2274	Combined stroke and death (30 days post-surgery)
Schmid_2017	2009-2014	non-RCT	Statutory German Quality Assurance Database, Germany	Mixed	80	22936/119138	Stroke (30 days post-surgery)	44948/96396	Stroke (30 days post-surgery)
						22936/119138	Death (30 days post-surgery	45678/96396	Death (30 days post-surgery)
						22936/119138	Combined stroke and death (30 days post-surgery)	45678/96396	Combined stroke and death (30 days post-surgery)
Schneider_1997	February 1985-March 1996	non-RCT	The Evanston and Glenbrook hospitals, USA	Mixed				155/271	Death (30 days post-surgery)
								155/271	Combined stroke and death (30 days post-surgery)
Schneider_2000	February 1985-January 1988	non-RCT	Evanston Hospital and Glenbrook Hospital, Illinois, USA	Mixed	80	90/492	Stroke (30 days post-surgery)		
						90/492	Death (30 days post-surgery		
						90/492	Combined stroke and death (30 days post-surgery)	91/492	Combined stroke and death (30 days post-surgery)

Author_Year	Recruitment period	Study design	Settings	Symptom	Age			Gender	
					Cut-off age	Sample size for analysis (Older/Younger)	Outcomes (periods)	Sample size for analysis (Female/Male)	Outcomes (periods)
Schultz_1988	NA	non-RCT	N/A	Mixed	80	105/116	Stroke (30 days post-surgery)		
						105/116	Death (30 days post-surgery		
						105/116	Combined stroke and death (30 days post-surgery)		
Sidawy_2009	-	non-RCT	56 centers in USA	Mixed				542/817	Stroke (30 days post-surgery)
								551/812	Death (30 days post-surgery)
								551/817	Combined stroke and death (30 days post-surgery)
Space_2006	March 2001-February 2006	RCT	35 centers in Germany, Austria, and Switzerland	Symptomatic	75	438/146	Combined stroke and death (30 days post-surgery)		
Stelagowski_2017	January 2004-August 2008	non-RCT	The department of Vascular, General and Oncologic Surgery, Copernicus Memorial Hospital in Łódź, Poland	Symptomatic				88/181	Combined stroke and death (30 days post-surgery)
Sternbach_2000	January 1992-September 1998	non-RCT	A single surgeon in an academic medical center in USA	Mixed				67/88	Stroke (30 days post-surgery)
								68/88	Death (30 days post-surgery)
								68/88	Combined stroke and death (30 days post-surgery)

Author_Year	Recruitment period	Study design	Settings	Symptom	Age			Gender	
					Cut-off age	Sample size for analysis (Older/Younger)	Outcomes (periods)	Sample size for analysis (Female/Male)	Outcomes (periods)
Stoner_2006	2000-2003	non-RCT	The National Surgical Quality Improvement Program (NSQIP) at the Veterans Affairs (VA) and the private sector (PS) databases, Colorado, USA	Mixed	80	1341/12281	Stroke (30 days post-surgery)		
						1341/12281	Death (30 days post-surgery	630/12992	Death (30 days post-surgery)
						1341/12281	Combined stroke and death (30 days post-surgery)	630/12992	Combined stroke and death (30 days post-surgery)
Teso_2004	1991-2002	non-RCT	St Mary's Hospital, London, UK	Mixed				5307/7318	Stroke (30 days post-surgery)
								5300/7318	Death (30 days post-surgery)
Teso_2005	1991-2001	non-RCT	St Mary's Hospital, London, UK	Mixed	80	1947/9365	Stroke (30 days post-surgery)		
						1947/9365	Death (30 days post-surgery		
Thomas_1996	1977-1994	non-RCT	Alfred Hospital Vascular Unit, Melbourne, Australia	Mixed	80	113/1705	Stroke (30 days post-surgery)		
						113/1705	Death (30 days post-surgery		
						113/1705	Combined stroke and death (30 days post-surgery)		
Thomson_2006	November 1994	non-RCT	-	Mixed				49/3942	Death (30 days post-surgery)

Author_Year	Recruitment period	Study design	Settings	Symptom	Age			Gender	
					Cut-off age	Sample size for analysis (Older/Younger)	Outcomes (periods)	Sample size for analysis (Female/Male)	Outcomes (periods)
Ting_2000	April 1993-September 1998	non-RCT	Vancouver General Hospital, Vancouver, Canada	Mixed	80	59/597	Stroke (30 days post-surgery)		
						59/597	Death (30 days post-surgery		
						59/597	Combined stroke and death (30 days post-surgery)		
TU_2003	January 1994-December 1997	non-RCT	The Ontario Carotid Endarterectomy Registry, Ontario, Canada	Symptomatic	75	1455/4583	Stroke (30 days post-surgery)	2035/3942	Stroke (30 days post-surgery)
						1455/4583	Death (30 days post-surgery	2096/3942	Death (30 days post-surgery)
						1455/4583	Combined stroke and death (30 days post-surgery)		
Van Damme_1996	1980-1994	non-RCT	2 University Hospitals, Belgium	Mixed	80	129/3732	Stroke (30 days post-surgery)		
						129/3732	Death (30 days post-surgery		
						129/3732	Combined stroke and death (30 days post-surgery)		
Voeks_2011	December 2000-July 2008	RCT	The Carotid Revascularization Endarterectomy vs Stenting Trial (CREST) (108 centers in USA and 9 centers in Canada)	Mixed	75	353/887	Stroke (30 days post-surgery)		
						353/887	Death (30 days post-surgery		
						353/887	Stroke (5 years post-surgery)		
						353/887	Death (5 years post-surgery		

Author_Year	Recruitment period	Study design	Settings	Symptom	Age			Gender	
					Cut-off age	Sample size for analysis (Older/Younger)	Outcomes (periods)	Sample size for analysis (Female/Male)	Outcomes (periods)
Weise_2004	-	non-RCT	-	Mixed				53/156	Stroke (30 days post-surgery)
