# Supplementary material for: Influences of age and gender on operative risks following carotid endarterectomy: A systematic review and meta-analysis
Source: PLoS One. 2023 May 10;18(5):e0285540. doi: 10.1371/journal.pone.0285540 (PMC10171679; doi:10.1371/journal.pone.0285540)
Supplement: S3 Table — (PDF) [file pone.0285540.s006.pdf]

S3 Table: Risk of bias

Author_Year	ROBIN-I							
	Bias due to confounding (baseline)	Bias selection of participants into the study	Bias in classification of interventions	Bias due to deviations from intended interventions	Bias due to missing data	Bias in measurement of outcomes	Bias in selection of the reported result	Interpretation
ACAS_1995	NI	L	L	L	L	L	L	NI
ACE_1999	NI	L	L	L	L	L	L	NI
Ackerstaff_2000	NI	L	L	L	L	L	L	NI
ACST_2004	NI	L	L	L	L	L	L	NI
Akbari_2000	S	L	L	L	L	L	L	S
Alozairi_2003	M	L	L	L	L	L	L	M
Alves-Ferreira_2019	NI	L	L	L	L	L	L	NI
Ascher_2001	NI	L	L	L	L	L	L	NI
Aune_2003	NI	NI	NI	NI	NI	NI	NI	NI
Ballotta_2000	S	L	L	L	L	L	L	S
Ballotta_2004	S	L	L	L	L	L	L	S
Bazan_2008	NI	L	L	L	L	L	L	NI
Blohme_1999	NI	L	L	L	L	L	L	NI
Brott (CREST)_2010	NI	L	L	L	L	L	L	NI
Brown_2008	NI	L	L	L	L	L	L	NI
Calvillo-king_2010	NI	L	L	L	L	L	L	NI
Cartier_2002	S	L	L	L	L	L	L	S

Author_Year	ROBIN-I							
	**Bias due to confounding (baseline)**	**Bias selection of participants into the study**	**Bias in classification of interventions**	**Bias due to deviations from intended interventions**	**Bias due to missing data**	**Bias in measurement of outcomes**	**Bias in selection of the reported result**	**Interpretation**
Cebul_1998	NI	L	L	L	L	L	L	NI
Coyle_1994	S	L	L	L	L	L	L	S
Dardik_2000	NI	L	L	L	L	L	L	NI
de Aguiar_2001	NI	L	L	L	L	L	L	NI
Debing_2007	S	L	L	L	L	L	L	S
de waard_2017	NI	L	L	L	L	L	L	NI
Djedovic_2018	S	L	L	L	L	L	L	S
Doonan_2019	S	L	L	L	L	L	L	S
Dorafshar_2004	L	L	L	L	L	L	L	L
Dorigo_2009	NI	L	L	L	L	L	L	NI
Dulai_2019	L	L	L	L	L	L	L	L
Eckstein_2002	NI	L	L	L	L	L	L	NI
ECST_1998	NI	L	L	L	L	L	L	NI
Ederle_2009	NI	L	L	L	L	L	L	NI
Fisher_1989	NI	L	L	L	L	L	L	NI
Frawley_2000	NI	L	L	L	L	L	L	NI
Friedmann_1988	NI	L	L	L	L	L	L	NI
Glousman_2020	S	L	L	L	L	L	L	S
Goldman_1999	L	L	L	L	L	L	L	L

Author_Year	ROBIN-I							
	Bias due to confounding (baseline)	Bias selection of participants into the study	Bias in classification of interventions	Bias due to deviations from intended interventions	Bias due to missing data	Bias in measurement of outcomes	Bias in selection of the reported result	Interpretation
Goldstein_1994	NI	L	L	L	L	L	L	NI
Goodney_2008	NI	L	L	L	L	L	L	NI
Grego_2005	NI	NI	NI	NI	NI	NI	NI	NI
Guzman_2014	L	L	L	L	L	L	L	L
Halm_2005	NI	L	L	L	L	L	L	NI
Halm_2009	NI	L	L	L	L	L	L	NI
Halliday (ACST 1)_2010	NI	L	L	L	L	L	L	NI
Harthun_2005	S	L	L	L	L	L	L	S
Hartmann_1999	NI	L	L	L	L	L	L	NI
Hertzer_1997	NI	L	L	L	L	L	L	NI
Hoffmann (BACASS)_2008	NI	L	L	L	L	L	L	NI
Hugh_2006	S	L	L	L	L	L	L	S
James_2001	S	L	L	L	L	L	L	S
Jeong_2019	L	L	L	L	L	L	L	L
Jim_2012	L	L	L	L	L	L	L	L
Jim_2014	L	L	L	L	L	L	L	L
Jordan_2002	NI	L	L	L	L	L	L	NI
Kang_2009	NI	L	L	L	L	L	L	NI
Kapral_2000	S	L	L	L	L	L	L	S
Kapral_2003	S	L	L	L	L	L	L	S

Author_Year	ROBIN-I							
	**Bias due to confounding (baseline)**	**Bias selection of participants into the study**	**Bias in classification of interventions**	**Bias due to deviations from intended interventions**	**Bias due to missing data**	**Bias in measurement of outcomes**	**Bias in selection of the reported result**	**Interpretation**
Karp_1998	NI	L	L	L	L	L	L	NI
Kazmers_1999	NI	L	L	L	L	L	L	NI
Kerdiles_1997	NI	NI	NI	NI	NI	NI	NI	NI
Khatri_2012	L	L	L	L	L	L	L	L
Knappich_2019	NI	L	L	L	L	L	L	NI
Kucey_1998	NI	L	L	L	L	L	L	NI
Lane_2003	L	L	L	L	L	L	L	L
Lau_2005	L	L	L	L	L	L	L	L
Love_2000	NI	L	L	L	L	L	L	NI
Lubke_2015	L	L	L	L	L	L	L	L
Magnadottir_1999	NI	L	L	L	L	L	L	NI
Magnan_1993	NI	L	L	L	L	L	L	NI
Mas (EVA-3S)_2006	NI	L	L	L	L	L	L	NI
Mattos_2001	S	L	L	L	L	L	L	S
Maxwell_1990	L	L	L	L	L	L	L	L
Maxwell_2000	S	L	L	L	L	L	L	S
Mazzalai_2009	NI	NI	NI	NI	NI	NI	NI	NI
Middleton_2002	NI	L	L	L	L	L	L	NI
Miller_2005	L	L	L	L	L	L	L	L
NASCET_1991	NI	L	L	L	L	L	L	NI

Author_Year	ROBIN-I							
	Bias due to confounding (baseline)	Bias selection of participants into the study	Bias in classification of interventions	Bias due to deviations from intended interventions	Bias due to missing data	Bias in measurement of outcomes	Bias in selection of the reported result	Interpretation
Navas_2008	NI	NI	NI	NI	NI	NI	NI	NI
Naylor_2000	NI	L	L	L	L	L	L	NI
Nunnelee_1995	L	L	L	L	L	L	L	L
Okawa_2015	L	L	L	L	L	L	L	L
Ommer_2001	S	L	L	L	L	L	L	S
Organ_2008	NI	L	L	L	L	L	L	NI
Ouriel_1986	NI	NI	NI	NI	NI	NI	NI	NI
Ozsvath_2002	L	L	L	L	L	L	L	L
Park_2008	L	L	L	L	L	L	L	L
Pasin_2019	NI	NI	NI	NI	NI	NI	NI	NI
Papachristou_1994	L	L	L	L	L	L	L	L
Perler_1996	NI	NI	NI	NI	NI	NI	NI	NI
Perler_1998	NI	L	L	L	L	L	L	NI
Pinkerton_1990	NI	L	L	L	L	L	L	NI
Pinkerton_2002	NI	L	L	L	L	L	L	NI
Plecha_1985	NI	L	L	L	L	L	L	NI
Plestis_1996	NI	L	L	L	L	L	L	NI
Pol_2013	S	L	L	L	L	L	L	S
Pruner_2003	S	L	L	L	L	L	L	S

Author_Year	ROBIN-I							
	**Bias due to confounding (baseline)**	**Bias selection of participants into the study**	**Bias in classification of interventions**	**Bias due to deviations from intended interventions**	**Bias due to missing data**	**Bias in measurement of outcomes**	**Bias in selection of the reported result**	**Interpretation**
Pulli_2005	NI	L	L	L	L	L	L	NI
Rajamani_2013	NI	L	L	L	L	L	L	NI
Rantner_2006	NI	L	L	L	L	L	L	NI
Reed_2003	NI	L	L	L	L	L	L	NI
Rigdon_1998	NI	NI	NI	NI	NI	NI	NI	NI
Riles_1994	NI	L	L	L	L	L	L	NI
Rockman_2001	S	L	L	L	L	L	L	S
Rockman_2003	S	L	L	L	L	L	L	S
Rong_2016	NI	L	L	L	L	L	L	NI
Salameh_2002	NI	L	L	L	L	L	L	NI
Salomon du Mont_2014	NI	L	L	L	L	L	L	NI
Sarac_2002	L	L	L	L	L	L	L	L
Schmid_2017	L	L	L	L	L	L	L	L
Schneider_1997	M	L	L	L	L	L	L	M
Schneider_2000	S	L	L	L	L	L	L	S
Schultz_1988	NI	NI	NI	NI	NI	NI	NI	NI
Sidawy_2009	NI	L	L	L	L	L	L	NI

Author_Year	ROBIN-I							
	Bias due to confounding (baseline)	Bias selection of participants into the study	Bias in classification of interventions	Bias due to deviations from intended interventions	Bias due to missing data	Bias in measurement of outcomes	Bias in selection of the reported result	Interpretation
Space_2006	NI	L	L	L	L	L	L	NI
Stelagowski_2017	S	L	L	L	L	L	L	S
Sternbach_2000	L	L	L	L	L	L	L	L
Stoner_2006	NI	L	L	L	L	L	L	NI
Teso_2004	L	L	L	L	L	L	L	L
Teso_2005	S	L	L	L	L	L	L	S
Thomas_1996	S	L	L	L	L	L	L	S
Thomson_2006	NI	NI	NI	NI	NI	NI	NI	NI
Ting_2000	NI	L	L	L	L	L	L	NI
TU_2003	NI	L	L	L	L	L	L	NI
Van Damme_1996	NI	NI	NI	NI	NI	NI	NI	NI
Voeks_2011	L	L	L	L	L	L	L	L
Weise_2004	S	L	L	L	L	L	L	S
Wong_1997	NI	L	L	L	L	L	L	NI

L, low risk of bias; NI, no information; S, serious risk of bias
