# Supplementary material for: Influences of age and gender on operative risks following carotid endarterectomy: A systematic review and meta-analysis
Source: PLoS One. 2023 May 10;18(5):e0285540. doi: 10.1371/journal.pone.0285540 (PMC10171679; doi:10.1371/journal.pone.0285540)
Supplement: S4 Table — (PDF) [file pone.0285540.s007.pdf]

S4 Table: Subgroup analyses of 30 days stroke, death, and combined stroke death risks of age and gender

Age									
Characteristics	30 days stroke			30 days death			30 days combined stroke and death		
	OR (95%CI)	Heterogeneity		OR (95%CI)	Heterogeneity		OR (95%CI)	Heterogeneity	
		I^2 value	P value		I^2 value	P value		I^2 value	P value
**Carotid stenosis symptoms**									
Symptomatic (≥80 vs <80)				1.17 [0.11,12.76]	59.2%	0.11			
Asymptomatic (≥80 vs <80)				2.44 [1.56,3.81]	-	-			
Mixed (≥80 vs <80)				1.77 [1.38,2.26]	37.5%	0.02			
**Gender**									
Characteristics	30 days stroke			30 days death			30 days combined stroke and death		
	OR (95%CI)	Heterogeneity		OR (95%CI)	Heterogeneity		OR (95%CI)	Heterogeneity	
		I^2 value	P value		I^2 value	P value		I^2 value	P value
**Carotid stenosis symptoms**									
Symptomatic (Female vs Male)	1.48 [1.07,2.04]	39.4%	0.17				1.26 [0.87,1.82]	47.1%	0.09
Asymptomatic (Female vs Male)	1.51 [1.14,1.99]	0.0%	0.72				1.50 [1.17,1.92]	0.0%	0.69
Mixed (Female vs Male)	1.23 [1.11,1.36]	30.6%	0.04				1.16 [1.04,1.30]	32.0%	0.03

Mixed, contained symptomatic and asymptomatic carotid stenoses;
