# Supplementary material for: Influences of age and gender on operative risks following carotid endarterectomy: A systematic review and meta-analysis
Source: PLoS One. 2023 May 10;18(5):e0285540. doi: 10.1371/journal.pone.0285540 (PMC10171679; doi:10.1371/journal.pone.0285540)
Supplement: S5 Table — (PDF) [file pone.0285540.s008.pdf]

S5 Table: Sensitivity analyses of 30 days stroke, death, and combined stroke death risks of age and gender

Age									
**Characteristics**	**30 days stroke**			**30 days death**			**30 days combined stroke and death**		
	**OR (95%CI)**	**Heterogeneity**		**OR (95%CI)**	**Heterogeneity**		**OR (95%CI)**	**Heterogeneity**	
		**I^2 value**	**P value**		**I^2 value**	**P value**		**I^2 value**	**P value**
**Removed studies affecting the influence plot** [removed Kang et al. 2009 and Schmid et al. 2017]									
Before removed (≥80 vs <80)				1.85 [1.48,2.30]	37.5%	0.02			
After removed (≥80 vs <80)				1.87 [1.53,2.29]	9.6%	0.32			
**Removed studies with unclear recruitment period** [removed Fisher et al. 1989 and Schultz et al. 1988]									
Before removed (≥80 vs <80)				1.85 [1.48,2.30]	37.5%	0.02			
After removed (≥80 vs <80)				1.82 [1.43,2.31]	41.4%	0.01			
**Gender**									
**Characteristics**	**30 days stroke**			**30 days death**			**30 days combined stroke and death**		
	**OR (95%CI)**	**Heterogeneity**		**OR (95%CI)**	**Heterogeneity**		**OR (95%CI)**	**Heterogeneity**	
		**I^2 value**	**P value**		**I^2 value**	**P value**		**I^2 value**	**P value**
**Removed studies affecting the influence plot** [removed ECST 1998 and Schmid et al. 2017]									
Before removed (Female vs Male)	1.28 [1.16,1.40]	34.7%	0.01				1.21 [1.09,1.34]	37.3%	0.00
After removed (Female vs Male)	1.26 [1.14,1.38]	16.5%	0.18				1.21 [1.10,1.33]	11.3%	0.26
**Removed studies with unclear recruitment period**									
Before removed (Female vs Male)	1.28 [1.16,1.40]	34.7%	0.01				1.21 [1.09,1.34]	37.3%	0.00
After removed (Female vs Male)	1.29 [1.16,1.43] [a]	38.9%	0.01				1.24 [1.11,1.38] [b]	36.9%	0.00

[a] Removed de Waard et al. 2017, Jim et al. 2014, Ranter et al. 2006, Rigdon et al. 1998, Sidawy et al. 2009, Weise et al. 2004

[b] Removed Knappich et al. 2019, Sidawy et al. 2009
