# Supplementary material for: Influences of age and gender on operative risks following carotid endarterectomy: A systematic review and meta-analysis
Source: PLoS One. 2023 May 10;18(5):e0285540. doi: 10.1371/journal.pone.0285540 (PMC10171679; doi:10.1371/journal.pone.0285540)
Supplement: S1 File — (PDF) [file pone.0285540.s009.pdf]

S1: References of included studies

Ackerstaff RGA, Moons KGM, van de Vlaskker CJW, Moll FL, Vermeulen FEE, Algra A, et al. Association of intraoperative transcranial doppler monitoring variables with stroke from carotid endarterectomy. Stroke 2000;31:1817-1823.

Alozairi O, MacKenzie RK, Morgan R, Cooper G, Engeset J, Brittenden J. Carotid endarterectomy in patients aged 75 and over: early results and late outcome. Eur J Vasc Endovasc Surg 2003;26:245-249.

Alves-Ferreira J, Rocha-Neves J, Dias-Neto M, Braga SF. Poor long-term outcomes after carotid endarterectomy: a retrospective analysis of two portuguese centers. Scand Cardiovasc J 2019;53:266-273.

Akbari CM, Pulling MC, Pomposelli FB Jr, Gibbons GW, Campbell DR, Logerfo FW. Gender and carotid endarterectomy: does it matter? J Vasc Surg 2000;31:1103-1108; discussion 1108-1109.

Ascher E, Hingorani A. Changing characteristics of carotid endarterectomy. Ann Vasc Surg 2001;15:275-280.

Aune S, Laxdal E, Pedersen G, Dregelid E. Patient characteristics, operative complications and long-term survival of patients aged 75 years or older subjected to carotid endarterectomy. Int Angiol 2003;22:421-425.

Ballotta E, Renon L, Da Giau G, Barbon B, Terranova O, Baracchini C. Octogenarians with contralateral carotid artery occlusion: a cohort at higher risk for carotid endarterectomy? J Vasc Surg 2004;39:1003-1008.

Ballotta E, Renon L, Da Giau G, Sarzo G, Abbruzzese E, Saladini M, et al. Carotid endarterectomy in women: early and long-term results. Surgery 2000;127:264-271.

Bazan HA, Pradhan S, Westvik TS, Sumpio BE, Gusberg RJ, Dardik A. Urgent carotid endarterectomy is safe in patients with few comorbid medical conditions. Ann Vasc Surg 2008;22:505-512.

Blohmé L, Sandström V, Hellström G, Swedenborg J, Takolander R. Complications in carotid endarterectomy are predicted by qualifying symptoms and preoperative CT findings. Eur J Vasc Endovasc Surg 1999;17:213-218.

Brott TG, Hobson RW, Howard G, Roubin GS, Clark WM, Brooks W, CREST Investigators. Stenting versus endarterectomy for treatment of carotid-artery stenosis. NEJM 2010;363(1):11-23.

Brown KE, Fanciullo DJ, Hicks T, Landau DS, Baraniewski HM, Morasch MD, et al. Carotid artery stenting compared to carotid endarterectomy performed exclusively in a veteran population: one center's experience with midterm results. Ann Surg 2008;248:110-116.

Calvillo-king L, Xuan L, Zhang S, Tuhrim S, Halm EA. Predicting risk of perioperative death and stroke after carotid endarterectomy in asymptomatic patients derivation and validation of a clinical risk score. Stroke 2010;41:2786-2794.

Cartier B. Carotid surgery in octogenarians: why not? Ann Vasc Surg 2002;16:751-755.

Cebul RD, Snow RJ, Pine R, Hertzer NR, Norris DG. Indications, outcomes, and provider volumes for carotid endarterectomy. JAMA 1998;279:1282-1287.

Coyle KA, Smith RB 3rd, Salam AA, Dodson TF, Chaikof EL, Lumsden AB. Carotid endarterectomy in the octogenarian. Ann Vasc Surg 1994;8:417-420.

Dardik A, Bowman HM, Gordon TA, Hsieh G, Perler BA. Impact of race on the outcome of carotid endarterectomy: a population-based analysis of 9,842 recent elective procedures. Ann Surg 2000;232:704-709.

Debing E, Van den Brande P. Carotid endarterectomy in the elderly: are the patient characteristics, the early outcome, and the predictors the same as those in younger patients? Surg Neurol 2007;67:467-471; discussion 471.

de Waard DD, de Borst GJ, Bulbulia R, Huibers A, Halliday A; Asymptomatic Carotid Surgery Trial-1 Collaborative Group. Diastolic blood pressure is a risk factor for peri-procedural stroke following carotid endarterectomy in asymptomatic patients. Eur J Vasc Endovasc Surg 2017;53:626-631.

Djedović M, Imširović B, Djedović S, Hadžimehmedagić A, Vukas H, Rovčanin B, et al. Carotid endarterectomy in women versus man: patient characteristics and perioperative complication (<30 Day). Open Access Maced J Med Sci 2018;6:463-466.

Doonan RJ, Abdullah A, Steinmetz-Wood S, Mekhaiel S, Steinmetz OK, Obrand DI, et al. Carotid endarterectomy outcomes in the elderly: a Canadian institutional experience. Ann Vasc Surg 2019;59:16-20.

Dorafshar AH, Reil TD, Moore WS, Quinones-Baldrich WJ, Angle N, Fahoomand F, et al. Cost analysis of carotid endarterectomy: is age a factor? Ann Vasc Surg 2004;18:729-735.

Dorigo W, Pulli R, Marek J, Troisi N, Pratesi G, Innocenti AA, et al. Carotid endarterectomy in female patients. J Vasc Surg 2009;50:1301-1307.

Dulai M, Tawfick W, Hynes N, Sultan S. Female gender as a risk factor for adverse outcomes after carotid revascularization. Ann Vasc Surg 2019;60:254-263.

Eckstein HH, Ringleb P, Dörfler A, Klemm K, Müller BT, Zegelman M, et al. The carotid surgery for ischemic stroke trial: a prospective observational study on carotid endarterectomy in the early period after ischemic stroke. J Vasc Surg 2002;36:997-1004.

Ederle J, Bonati LH, Dobson J, Featherstone RL, Gaines PA, Beard JD, et al; CAVATAS Investigators. Endovascular treatment with angioplasty or stenting versus endarterectomy in patients with carotid artery stenosis in the Carotid and Vertebral Artery Transluminal Angioplasty Study (CAVATAS): long-term follow-up of a randomised trial. Lancet Neurol 2009;8:898-907.

European Carotid Surgery Trialists' Collaborative Group. Randomised trial of endarterectomy for recently symptomatic carotid stenosis: final results of the MRC European Carotid Surgery Trial (ECST). Lancet 1998;351:1379-1387.

Executive Committee for the Asymptomatic Carotid Atherosclerosis Study (ACAS). Endarterectomy for asymptomatic carotid artery stenosis. JAMA 1995;273:1421-1428.

Fisher ES, Malenka DJ, Solomon NA, Bubolz TA, Whaley FS, Wennberg JE. Risk of carotid endarterectomy in the elderly. Am J Public Health 1989;79:1617-1620.

Frawley JE, Hicks RG, Woodforth IJ. Risk factors for peri-operative stroke complicating carotid endarterectomy: selective analysis of a prospective audit of 1000 consecutive operations. Aust N Z J Surg 2000;70:52-56.

Friedmann P, Garb JL, Berman J, Sullivan C, Celoria G, Rhee SW. Carotid endarterectomy. Clinical results in a community-based teaching hospital. Stroke 1988;19:1323-1327.

Glousman BN, Sebastian R, Macata R, Kuang X, Yang A, Patel D, et al. Carotid endarterectomy for asymptomatic carotid stenosis is safe in octogenarians. J Vasc Surg 2020;71:518-524.

Goldman KA, Singhal A, Kahn SP, Davidson JT, Patel N, Patel T, et al. Carotid Artery Endarterectomy in the Octogenarian: A Community Hospital Experience. Vascular Surgery 1999;33:451-460.

Goldstein LB, McCrory DC, Landsman PB, Samsa GP, Ancukiewicz M, Oddone EZ, et al. Multicenter review of preoperative risk factors for carotid endarterectomy in patients with ipsilateral symptoms. Stroke 1994;25:1116-1121.

Goodney PP, Likosky DS, Cronenwett JL; Vascular Study Group of Northern New England. Factors associated with stroke or death after carotid endarterectomy in Northern New England. J Vasc Surg 2008;48:1139-1145.

Grego F, Lepidi S, Antonello M, Bonvini S, Battocchio P, Galzignan E, et al. Is carotid endarterectomy in octogenarians more dangerous than in younger patients? J Cardiovasc Surg (Torino) 2005;46:477-483.

Guzman RP, Weighell W, Guzman C, Rodriguez-Leyva D. Female sex does not influence 30-day stroke and mortality rates after carotid endarterectomy. Ann Vasc Surg 2014;28:245-252.

Halliday A, Harrison M, Hayter E, Kong X, Mansfield A, Marro J, Asymptomatic Carotid Surgery Trial (ACST) Collaborative Group. 10-year stroke prevention after successful carotid endarterectomy for asymptomatic stenosis (ACST-1): a multicentre randomised trial. Lancet 2010;376(9746):1074-84.

Halm EA, Hannan EL, Rojas M, Tuhrim S, Riles TS, Rockman CB, et al. Clinical and operative predictors of outcomes of carotid endarterectomy. J Vasc Surg 2005;42:420-428.

Halm EA, Tuhrim S, Wang JJ, Rockman C, Riles TS, Chassin MR. Risk factors for perioperative death and stroke after carotid endarterectomy: results of the new york carotid artery surgery study. Stroke 2009;40:221-229.

Harthun NL, Kongable GL, Baglioni AJ, Meakem TD, Kron IL. Examination of sex as an independent risk factor for adverse events after carotid endarterectomy. J Vasc Surg 2005;41:223-230.

Hartmann A, Hupp T, Koch HC, Dollinger P, Stapf C, Schmidt R, et al. Prospective study on the complication rate of carotid surgery. Cerebrovasc Dis 1999;9:152-156.

Hertzer NR, O'Hara PJ, Mascha EJ, Krajewski LP, Sullivan TM, Beven EG. Early outcome assessment for 2228 consecutive carotid endarterectomy procedures: the Cleveland Clinic experience from 1989 to 1995. J Vasc Surg 1997;26:1-10.

Hoffmann A, Engelter S, Taschner C, Mendelowitsch A, Merlo A, Radue EW, et al. Carotid artery stenting versus carotid endarterectomy – a prospective randomised controlled single-centre trial with long-term follow up (BACASS). Schweizer Archiv für Neurologie und Psychiatrie 2008;159:84-9.

Hugl B, Oldenburg WA, Neuhauser B, Hakaim AG. Effect of age and gender on restenosis after carotid endarterectomy. Ann Vasc Surg 2006;20:602-608.

International Carotid Stenting Study investigators. Carotid artery stenting compared with endarterectomy in patients with symptomatic carotid stenosis (International Carotid Stenting Study): an interim analysis of a randomised controlled trial. Lancet 2010;375(9719):985-97.

James DC, Hughes JD, Mills JL, Westerband A. The influence of gender on complications of carotid endarterectomy. Am J Surg 2001;182:654-657.

Jeong MJ, Kwon SU, Kim MJ, Han Y, Kwon TW, Cho YP. Effects of patient age on outcomes after carotid endarterectomy: a retrospective, single-center study in Korea. Medicine (Baltimore) 2019;98:e16781. doi: 10.1097/MD.0000000000016781.

Jim J, Dillavou ED, Upchurch GR Jr, Osborne NH, Kenwood CT, Siami FS, et al. Gender-specific 30-day outcomes after carotid endarterectomy and carotid artery stenting in the Society for Vascular Surgery Vascular Registry. J Vasc Surg 2014;59:742-748.

Jim J, Rubin BG, Ricotta JJ, Kenwood CT, Siami FS, Sicard GA. SVS Outcomes Committee. Society for Vascular Surgery (SVS) Vascular Registry evaluation of comparative effectiveness of carotid revascularization procedures stratified by Medicare age. J Vasc Surg 2012;55:1313-1321.

Jordan WD, Alcocer F, Wirthlin DJ, Warren JA, McDowell HA, Whitley WD, et al. High-risk carotid endarterectomy: Challenges for carotid stent protocols. J Vasc Surg 2002;35:16-22.

Kang JL, Chung TK, Lancaster RT, LaMuraglia GM, Conrad MF, Cambria RP. Outcomes after carotid endarterectomy: Is there a high-risk population? A National Surgical Quality Improvement Program report. J Vasc Surg 2009;49:331-339.

Kapral MK, Redelmeier DA. Carotid endarterectomy for women and men. J Womens Health Gend Based Med. 2000;9:987-994.

Kapral MK, Wang H, Austin PC, Fang J, Kucey D, Bowyer B, et al. Sex differences in carotid endarterectomy outcomes: results from the Ontario Carotid Endarterectomy Registry. Stroke 2003;34:1120-1125.

Karp HR, Flanders WD, Shipp CC, Taylor B, Martin D. Carotid endarterectomy among medicare beneficiaries: a statewide evaluation of appropriateness and outcome. Stroke 1998;29:46-52.

Kazmers A, Perkins AJ, Huber TS, Jacobs LA. Carotid surgery in octogenarians in Veterans Affairs medical centers. J Surg Res 1999;81:87-90.

Kerdiles Y, Lucas A, Podeur L, Ferte P, Cardon A. Results of carotid surgery in elderly patients. J Cardiovasc Surg (Torino) 1997;38:327-334.

Khatri R, Chaudhry SA, Vazquez G, Rodriguez GJ, Hassan AE, Suri MFK, et al. Age differential between outcomes of carotid angioplasty and stent placement and carotid endarterectomy in general practice. J Vasc Surg 2012;55:72-78.

Knappich C, Kuehnl A, Haller B, Salvermoser M, Algra A, Becquemin JP, et al. Associations of perioperative variables with the 30-day risk of stroke or death in carotid endarterectomy for symptomatic carotid stenosis. Stroke 2019;50:3439-3448.

Kucey DS, Bowyer B, Iron K, Austin P, Anderson G, Tu JV. Determinants of outcome after carotid endarterectomy. J Vasc Surg 1998;28:1051-1058.

Lane JS, Shekherdimian S, Moore WS. Does female gender or hormone replacement therapy affect early or late outcome after carotid endarterectomy? J Vasc Surg 2003;37:568-574.

Lau D, Granke K, Olabisi R, Basson MD, Vouyouka A. Carotid endarterectomy in octogenarian veterans: does age affect outcome? a single-center experience. Am J Surg 2005;190:795-799.

Love A, Hollyoak MA. Carotid endarterectomy and local anaesthesia: reducing the disasters. Cardiovasc Surg 2000;8:429-435.

Lubke T, Ahmad W, Jalali BK, Brunkwall J. Gender-based 30-day and long-term outcomes after carotid endarterectomy. VASA 2015;44:289-295.

Magnadottir HB, Lightdale N, Harbaugh RE. Clinical outcomes for patients at high risk who underwent carotid endarterectomy with regional anesthesia. Neurosurgery 1999;45:786-792.

Magnan PE, Caus T, Branchereau A, Rosset E, Prima F, France M. Internal carotid artery surgery: ten-year results. Ann Vasc Surg 1993;7:521-529.

Mas JL, Chatellier G, Beyssen B, Branchereau A, Moulin T, Becquemin JP, EVA-3S Investigators. Endarterectomy versus stenting in patients with symptomatic severe carotid stenosis. NEJM 2006;355(16):1660-71.

Mattos MA, Sumner DS, Bohannon WT, Parra J, McLafferty RB, Karch LA, Ramsey DE, Hodgson KJ. Carotid endarterectomy in women: challenging the results from ACAS and NASCET. Ann Surg 2001;234:438-445; discussion 445-446.

Maxwell JG, Rutherford EJ, Covington DL, Churchill P, Patrick RD, Scott C, et al. Community hospital carotid endarterectomy in patients over age 75. Am J Surg 1990;160:598-603.

Maxwell JG, Taylor AJ, Maxwell BG, Brinker CC, Covington DL, Tinsley E Jr. Carotid endarterectomy in the community hospital in patients age 80 and older. Ann Surg 2000;231:781-788.

Mazzalai F, Terranova O, Gruppo M, Meneghetti G, Baracchini C, Ballotta E. Octogenarians and nonagenarians with severe symptomatic and asymptomatic carotid disease: does older age indicate "high risk" for carotid endarterectomy?. BMC Geriatr 2009;9. doi: 10.1186/1471-2318-9-S1-A4.

Middleton S, Donnelly N; New South Wales Carotid Endarterctomy Audit Project Working Group. Outcomes of carotid endarterectomy: how does the Australian state of New South Wales compare with international benchmarks? J Vasc Surg 2002;36:62-69.

Miller MT, Comerota AJ, Tzilinis A, Daoud Y, Hammerling J. Carotid endarterectomy in octogenarians: does increased age indicate "high risk?". J Vasc Surg 2005;41:231-237.

MRC Asymptomatic Carotid Surgery Trial (ACST) Collaborative Group. Prevention of disabling and fatal strokes by successful carotid endarterectomy in patients without recent neurological symptoms: randomised controlled trial. Lancet 2004;363:1491-1502.

North American Symptomatic Carotid Endarterectomy Trial Collaborative Group. Beneficial effect of carotid endarterectomy in symptomatic patients with high-grade carotid stenosis. *NEJM* 1991;325(7):445-53.

Navas Vinagre I, Royo Serrando J, Dolz Jordi JL, Salazar Tortolero G, Fragoso Martínez M, Escalante Arroyo S, et al. Carotid thromboendarterectomy in the elderly patient: clinical characteristics in operated patients and short and middle term results. Neurologia 2008;23:408-414.

Naylor AR, Hayes PD, Allroggen H, Lennard N, Gaunt ME, Thompson MM, et al. Reducing the risk of carotid surgery: a 7-year audit of the role of monitoring and quality control assessment. J Vasc Surg 2000;32:750-759.

Nunnelee JD, Kurgan A, Auer AI. Carotid endarterectomy in elderly vascular patients: experience in a community hospital. Geriatr Nurs 1995;16:121-123.

Okawa M, Ogata T, Abe H, Fukuda K, Higashi T, Inoue T. Do octogenarians still have a high risk of adverse outcomes after carotid endarterectomy in the era of a super-aged society? a single-center study in Japan. J Stroke Cerebrovasc Dis 2015;24:370-373.

Ommer A, Pillny M, Grabitz K, Sandmann W. Reconstructive surgery for carotid artery occlusive disease in the elderly--a high risk operation? Cardiovasc Surg 2001;9:552-558.

Organ N, Walker PJ, Jenkins J, Foster W, Jenkins J. 15 year experience of carotid endarterectomy at the Royal Brisbane and Women's Hospital: outcomes and changing trends in management. Eur J Vasc Endovasc Surg 2008;35:273-279.

Ouriel K, Penn TE, Ricotta JJ, May AG, Green RM, DeWeese JA. Carotid endarterectomy in the elderly patient. Surg Gynecol Obstet 1986;162:334-336.

Ozsvath KJ, Darling RC, Tabatabai L, Roddy SP, Paty PS, Chang BB, et al. Carotid endarterectomy in the elderly: does gender effect outcome? Cardiovasc Surg 2002;10:534-537.

Papachristou EA, Dragojevic D. Surgical procedures of the carotid system in the elderly. Vascular Surgery1994;28:531-537.

Park B, Aiello F, Dahn MS, Menzoian JO, Mavanur A. No gender influences on clinical outcomes or durability of repair following carotid angioplasty with stenting and carotid endarterectomy. Vasc Endovascular Surg 2008;42:321-328.

Pasin L, Marrocco Trischitta MM, Landoni G, Piras D, Nardelli P, Cornero G, et al. Operative morbidity and mortality in octogenarians after carotid endarterectomy: a propensity score matching study. J Cardiovasc Surg (Torino) 2019;60:703-707.

Perler BA. The impact of advanced age on the results of carotid endarterectomy: an outcome analysis. J Am Coll Surg 1996;183:559-564.

Perler BA, Dardik A, Burleyson GP, Gordon TA, Williams GM. Influence of age and hospital volume on the results of carotid endarterectomy: a statewide analysis of 9918 cases. J Vasc Surg 1998;27:25-31; discussion 31-33.

Pinkerton JA Jr, Gholkar VR. Should patient age be a consideration in carotid endarterectomy? J Vasc Surg 1990;11:650-658.

Pinkerton JA Jr. EEG as a criterion for shunt need in carotid endarterectomy. Ann Vasc Surg 2002;16:756-761.

Plecha FR, Bertin VJ, Plecha EJ, Avellone JC, Farrell CJ, Hertzer NR, et al. The early results of vascular surgery in patients 75 years of age and older: an analysis of 3259 cases. J Vasc Surg 1985;2:769-774.

Plestis KA, Kantis G, Haygood K, Earl N, Howell JF. Carotid endarterectomy with homologous vein patch angioplasty: a review of 1006 cases. J Vasc Surg 1996;24:109-119.

Pol RA, Reijnen MM, Lont M, Tielliu IF, van Sterkenburg SM, van den Dungen JJ, et al. Safety and efficacy of carotid endarterectomy in octogenarians. Ann Vasc Surg 2013;27:736-742.

Pruner G, Castellano R, Jannello Am AM, Astore D, Civilini E, Melissano G, et al. Carotid endarterectomy in the octogenarian: outcomes of 345 procedures performed from 1995-2000. Cardiovasc Surg 2003;11:105-112.

Pulli R, Dorigo W, Barbanti E, Azas L, Pratesi G, Innocenti AA, et al. Does the high-risk patient for carotid endarterectomy really exist? Am J Surg 2005;189:714-719.

Rajamani K, Kennedy KF, Ruggiero NJ, Rosenfield K, Spertus J, Chaturvedi S. Outcomes of carotid endarterectomy in the elderly: report from the National Cardiovascular Data Registry. Stroke 2013;44:1172-1174.

Rantner B, Eckstein HH, Ringleb P, Woelfle KD, Bruijnen H, Schmidauer C, et al. American Society of Anesthesiology and Rankin as predictive parameters for the outcome of carotid endarterectomy within 28 days after an ischemic stroke. J Stroke Cerebrovasc Dis 2006;15:114-120.

Reed AB, Gaccione P, Belkin M, Donaldson MC, Mannick JA, Whittemore AD, et al. Preoperative risk factors for carotid endarterectomy: defining the patient at high risk. J Vasc Surg 2003;37:1191-1199.

Rigdon EE. Racial and gender differences in outcome after carotid endarterectomy. Am Surg 1998;64:527-530; discussion 530-532.

Riles TS, Imparato AM, Jacobowitz GR, Lamparello PJ, Giangola G, Adelman MA, et al. The cause of perioperative stroke after carotid endarterectomy. J Vasc Surg 1994;19:206-214; discussion 215-216.

Rockman CB, Castillo J, Adelman MA, Jacobowitz GR, Gagne PJ, Lamparello PJ, et al. Carotid endarterectomy in female patients: are the concerns of the Asymptomatic Carotid Atherosclerosis Study valid? J Vasc Surg 2001;33:236-240; discussion 240-241.

Rockman CB, Jacobowitz GR, Adelman MA, Lamparello PJ, Gagne PJ, Landis R, et al. The benefits of carotid endarterectomy in the octogenarian: a challenge to the results of carotid angioplasty and stenting. Ann Vasc Surg 2003;17:9-14.

Rong X, Yang W, Garzon-Muvdi T, Ye X, Caplan JM, Colby GP, et al. Risk factors associated with ipsilateral ischemic events following carotid endarterectomy for carotid artery stenosis. World Neurosurg 2016;89:611-619.

Salameh JR, Myers JL, Mukherjee D. Carotid endarterectomy in elderly patients: low complication rate with overnight stay. Arch Surg 2002;137:1284-1287; discussion 1288.

Salomon du Mont L, Ravelojaona M, Puyraveau M, Al Sayed M, Ritucci E, Rinckenbach S. Carotid endarterectomy in octogenarian: short- and midterm results. Ann Vasc Surg 2014;28:917-923.

Sarac TP, Hertzer NR, Mascha EJ, O'Hara PJ, Krajewski LP, Clair DG, et al. Gender as a primary predictor of outcome after carotid endarterectomy. J Vasc Surg 2002;35:748-753.

Schmid S, Tsantilas P, Knappich C, Kallmayer M, König T, Breitkreuz T, et al. Risk of inhospital stroke or death is associated with age but not sex in patients treated with carotid endarterectomy for asymptomatic or symptomatic stenosis in routine practice: secondary data analysis of the Nationwide German Statutory Quality Assurance Database From 2009 to 2014. J Am Heart Assoc 2017;6. doi: 10.1161/JAHA.116.004764.

Schneider JR, Droste JS, Golan JF. Carotid endarterectomy in women versus men: patient characteristics and outcomes. J Vasc Sur 1997;25:890-896; discussion 897-898.

Schneider JR, Droste JS, Schindler N, Golan JF. Carotid endarterectomy in octogenarians: comparison with patient characteristics and outcomes in younger patients. J Vasc Surg 2000;31:927-935.

Schultz RD, Sterpetti AV, Feldhaus RJ. Carotid endarterectomy in octogenarians and nonagenarians. Surg Gynecol Obstet 1988;166:245-251.

Sidawy AN, Zwolak RM, White RA, Siami FS, Schermerhorn ML, Sicard GA, et al. Risk-adjusted 30-day outcomes of carotid stenting and endarterectomy: results from the SVS Vascular Registry. J Vasc Surg 2009;49:71-79.

SPACE Collaborative Group, Ringleb PA, Allenberg J, Brückmann H, Eckstein HH, Fraedrich G, et al. 30 day results from the SPACE trial of stent-protected angioplasty versus carotid endarterectomy in symptomatic patients: a randomised non-inferiority trial. Lancet 2006;368:1239-1247.

Stelągowski M, Kasielska-Trojan A, Bogusiak K, Timler D, Łysakowski M, Kaźmierski P. Gender-related risk factors for perioperative stroke after carotid endarterectomy in symptomatic patients. Adv Clin Exp Med 2017;26:1225-1231.

Sternbach Y, Perler BA. The influence of female gender on the outcome of carotid endarterectomy: a challenge to the ACAS findings. Surgery 2000;127:272-275.

Stoner MC, Abbott WM, Wong DR, Hua HT, LaMuraglia GM, Kwolck CJ, et al. Defining the high-risk patient for carotid endarterectomy: an analysis of the prospective national surgical quality improvement program database. J Vasc Surg 2006;43:285-296.

Taylor DW, Barnett HJM, Haynes RB, Ferguson GG, Sackett DL, Thorpe KE, et al. Low-dose and high-dose acetylsalicylic acid for patients undergoing carotid endarterectomy: a randomised controlled trial. Lancet 1999;353:2179-2184.

Teso D, Edwards RE, Antezana JN, Dudrick SJ, Dardik A. Do vascular surgeons improve the outcome of carotid endarterectomy? an analysis of 12,618 elective cases in the state of Connecticut. Vascular 2004;12:155-165.

Teso D, Edwards RE, Frattini JC, Dudrick SJ, Dardik A. Safety of carotid endarterectomy in 2,443 elderly patients: lessons from nonagenarians--are we pushing the limit? J Am Coll Surg 2005;200:734-741.

Thomas PC, Grigg M. Carotid artery surgery in the octogenarian. Aust N Z J Surg 1996;66:231-234.

Thomson I, Fanous M. Carotid endarterectomy under regional anaesthesia at Dunedin Hospital, New Zealand: 1994-2003. N Z Med J 2006;119(1230):U1882.

Ting AC, Taylor DC, Salvian AJ, Chen JC, Strandberg S, Hsiang YN. Carotid endarterectomy in octogenerians. Cardiovasc Surg 2000;8:441-445.

Toledo de Aguiar E, Lederman A, Higutchi C, Schreen G. Early and late results of carotid endarterectomy: retrospective study of 70 operations. Sao Paulo Med J 2001;119:206-211.

Tu JV, Wang H, Bowyer B, Green L, Fang J, Kucey D, et al. Risk factors for death or stroke after carotid endarterectomy: observations from the Ontario Carotid Endarterectomy Registry. Stroke 2003;34:2568-2573.

Van Damme H, Lacroix H, Desiron Q, Nevelsteen A, Limet R, Suy R. Carotid surgery in octogenarians: is it worthwhile? Acta Chir Belg 1996;96:71-77.

Voeks JH, Howard G, Roubin GS, Malas MB, Cohen DJ, Sternbergh WC, et al.; CREST Investigators. Age and outcomes after carotid stenting and endarterectomy: the carotid revascularization endarterectomy versus stenting trial. Stroke 2011;42:3484-3490.

Weise J, Kuschke S, Bähr M. Gender-specific risk of perioperative complications in carotid endarterectomy patients with contralateral carotid artery stenosis or occlusion. J Neurol 2004;251:838-844.

Wong JH, Findlay JM, Suarez-Almazor ME. Hemodynamic instability after carotid endarterectomy: risk factors and associations with operative complications. Neurosurgery 1997;41:35-41; discussion 41-43
