# Supplementary material for: Influences of age and gender on operative risks following carotid endarterectomy: A systematic review and meta-analysis
Source: PLoS One. 2023 May 10;18(5):e0285540. doi: 10.1371/journal.pone.0285540 (PMC10171679; doi:10.1371/journal.pone.0285540)
Supplement: Attachment — Submitted filename: Response to Reviewers_R1.docx [file pone.0285540.s010.docx]

**RESPONSES TO REVIEWER'S COMMENTS: PONE-D-22-30639**

**“Influences of age and gender on operative risks following carotid endarterectomy: a systematic review and meta-analysis”**

**Journal Requirements**

**Please ensure that your manuscript meets PLOS ONE's style requirements, including those for file naming.**

Reply: We have corrected all to be met the journal’s style.

**We note that this manuscript is a systematic review or meta-analysis; our author guidelines therefore require that you use PRISMA guidance to help improve reporting quality of this type of study. Please upload copies of the completed PRISMA checklist as Supporting Information with a file name “PRISMA checklist”.**

Reply: The requested file has been prepared and submitted as separated file.

**Review #1**

**Many thanks for the opportunity to review this manuscript reporting the results of a meta-analysis of studies reporting age and/or sex associations with outcomes following carotid endarterectomy. This is a large and apparently rigorous review of available literature which has included and pooled data from a large number of studies. Whilst this offers breadth, my main concern with the methodology of this meta-analysis is heterogeneity, particularly in the meta-analysis of associations of age with outcomes. Whilst I commend the authors on their work, I think major revisions are required to the analyses before I could recommend this manuscript for publication. Please find detailed comments below:**

Reply: We are thankful for dedicating your valuable time to feedback our work and provide the very useful comments to us. These comments would help to improve our manuscript quality.

**1. It is very rare that fixed-effect analyses are suitable. I do not think fixed-effects analyses are appropriate in this type of meta-analysis. The authors have used a mix of observational and randomised data and as such the heterogeneity of study designs is too great for fixed-effects analyses to be used. It is also not clear from the reporting which meta-analyses the authors have performed have used a fixed-effects model. The authors should repeat any analyses where a fixed-effects model has been used and instead use a random effects model.**

Reply: You raise a very important point. We totally agree with you that fixed-effects model is not suitable for this meta-analysis type. Previously, we performed fixed-effects model in some analysis containing less heterogeneity, based on *I*^2^. However, we re-considered that random effects model would be more appropriate rather than fixed-effects model. This is also because most analyses contained high heterogeneity. Even in less heterogeneity, we should carry out random effects model due to missed and different types of study design. Thus, we re-analyzed using random effects model throughout the analyses. We revised it within the following sections;

- Abstract: Result part (p.3-4, lines 62-70)
- Methods: Statistical analyses part (p.8, lines 169-170)
- Table 1 (p.10, line 202)
- Results: Effect of age on stroke, death, and combined stroke and death… (p.13-14, lines 215-235), Effect of gender on….(p.16-17, lines 261-277, 290-293)
- Figs 1-4
- S1 Fig. Other main results (S1_Fig_R1)
- S4 Table. Subgroup analyses (S4_Table_R1)
- S5 Table. Sensitivity analyses (S5_Table_R1)

**2. Whilst I accept that the use of I^2 as a measure of heterogeneity is widely used in the literature, it is frequently used and interpreted inappropriately (**[**https://bmcmedresmethodol.biomedcentral.com/articles/10.1186/1471-2288-8-79**](https://bmcmedresmethodol.biomedcentral.com/articles/10.1186/1471-2288-8-79)**). It definitely should not be used to decide on whether a fixed- or random-effects analysis should be used. As above, please use a random-effects model for all analyses. Consider reporting Tau^2 in addition to I^2 as this represents the ammount of between-study heterogeneity whilst I^2 estimates the % of variability due to between-study heterogeneity and precision of the effect estimates has a big impact on I^2.**

Reply: Thank you for the useful suggestion. We re-considered using random effects model in all analyses as you suggested. All revised sections were the same as the previous question.

**3. I do not think the meta-analyses of age in its current form is valid. At the very least the forest plot should be presented in subgroups of studies that compared ages at different cut points (<65 and 65+; <70 and 70+; <75 and 75+; <80 and 80+). However I'm not sure that pooling all these studies with different cut points is appropriate as this pools data from different age cohorts. The most robust way to investigate the influence of age would be to perform separate meta-analyses pooling data from studies that have used the same cut-point only.**

Reply: We thank the valid comments as we should not pool the studies with different cut points of age. We have re-arranged and re-analysed by grouping the same age for meta-analysis as follows; age >=75 vs <75; >=80 vs <80. For other age, we could not perform meta-analysis due to insufficient number of studies. We have now amended this within;

- Abstract: Result part (p.3-4, lines 62-70)
- Table 1 (p.10, line 202)
- Results: Effect of age on stroke, death, and combined stroke and death… (p.13-14, lines 215-235)
- Figures 1-3
- S1 Fig. Other main results (S1_Fig_R1)
- S4 Table. Subgroup analyses (S4_Table_R1)
- S5 Table. Sensitivity analyses (S5_Table_R1)

**4. Similarly I have concerns about the meta-analyses of short- and long-term outcomes. The time points for these outcomes are not defined in the methods. Given that the outcomes are reported as odds ratios then the outcome measures themselves need to be measured at the same timepoint (e.g. 30 day mortality pooled together, 1 year mortality pooled together, 5 year mortality pooled together). Studies reporting outcome measures at different timepoints should not be pooled in the same analysis.**

Reply: Short-term outcome is defined as an outcome measured at 30 days after surgery, while long-term outcome is defined as an outcome measured at the time up to 5 years post-surgery. We have clarified this in the Method section under ‘Statistical analyses’ (p.8, lines 168-169). We also agreed with you that the pooled meta-analysis should be performed in studies reporting outcomes measured at the same time point. We have re-analysed and revised findings within;

- Abstract: Result part (p.3-4, lines 62-70)
- Table 1 (p.10, line 202)
- Results: Effect of age on stroke, death, and combined stroke and death… (p.13-14, lines 215-235), Effect of gender on….(p.16-17, lines 261-277, 290-293)
- Figs 1-4
- S1 Fig. Other main results (S1_Fig_R1)
- S4 Table. Subgroup analyses (S4_Table_R1)
- S5 Table. Sensitivity analyses (S5_Table_R1)

**5. There is a notable lack of table summarising included studies presented in the manuscript or supplementary material. The authors state all the data that has been extracted but this is not presented anywhere. I think there should be a table included which describes the data included. Of particular note, there is no information of sample size for each individual study presented anywhere in either the manuscript or supplementary material. A table presenting the raw data extracted from each study as detailed in the methodology and also including the sample size should be presented in the supplementary material. This would provide important context to enable readers to interpret the results appropriately.**

Reply: The reviewer raises the valid point that should have been identified. We have added a table of characteristics of included studies as shown in S2 Table. Characteristics of included studies (S2_Table_R1).

**6. Are the authors confident that there is no double-counting of participants? For example, have more than one study utilised data from the same registry e.g. ACS-NSQIP, VSQIP, NVR etc. The authors need to take care not to include studies that have utilised the same database with overlapping timeframes in the same meta-analysis as this risks potentially including the same participants and outcome events in the same meta-analysis multiple times. This risk is highlighted in this recent paper by Hussein et al (**[**https://bmcpublichealth.biomedcentral.com/articles/10.1186/s12889-022-14213-6**](https://bmcpublichealth.biomedcentral.com/articles/10.1186/s12889-022-14213-6)**).**

Reply: Thank you for the suggestion. We have re-checked studies using same databases and identified overlapped recruitment period. As for one of registries above, we have clarified that there are three studies using ACS-NSQIP (Goldman 2020, Kang 2009, Stoner 2006). However, these studies identified different timeframe. Also, these studies reported 30 days operative risk after surgery, which reflects reported new cases. Thus, the different timeframe and reported new cases support our confidence to keep these studies in the meta-analysis. For any studies using same registry with same timeframe were excluded. We have revised as follows number of studies for meta-analysis in;

- Figure 1 PRISMA flow chart
- Abstract section under ‘Results’ (p.3, lines 59-61)
- Result section (p.9, lines 189-196)

**7. I presume all of the data extracted and pooled are unadjusted for confounders. If so, this is a significant limitation to the study and should be mentioned. There is a very high risk that any observed effect is due to confounding in the individual studies and that pooling data magnifies this effect. It would be interesting, if possible, to include a meta-analysis of suitable outcome measures using effect estimates from studies that used comparible adjutment models to reduce the impact of confounding. This would also have limitations (especially as it is unlikely adjustment models were the same between studies) but may increase the confidence of findings.**

Reply: The reviewer raised a valid point that very important to our study. We totally agree with you that it would be a significant limitation to our study due to extracting all unadjusted values. This is not a systematic review based on individual patients data (IPD) set. We extracted summary data from study publications rather than the original research data, which are sought directly from the researchers responsible for each study. Therefore, we cannot adjust for confounders.

We could not extract adjusted Odds Ratios in each included study as those studies’ objective were not primarily investigate association between age/ or gender and CEA on outcomes (stroke, death, combined stroke and death). For example, a study aimed to compare stroke event between two surgical procedures (CEA vs stent). In CEA, the stroke event was additionally identified in subgroup of age and gender. An adjusted Odd Ratio, if any, would only be available for the primary aim. Thus, we could only pick up event, non-event, and total number of patients for calculating Odd Ratio and pooled effect estimates for meta-analysis instead. However, we have added this important point as a limitation of the study. This has been clarified in the Strength and imitations section (p.21, lines 389-392). The additional statement is as follow.

*‘Third, pooled effect estimates of this meta-analysis with unadjusted confounders are accounted as a significant limitation. As age and gender among most identified studies were not the main determinant of interest, we could not extract the adjusted effects. Thus, the meta-analysis finding should be interpreted with caution.’*

**8. I am unclear from the methods and reporting of results what sensitivity analyses have been performed. Subgroup analyses are usually presented as a single forest plot divided into relevant subgroups. Sensitivity analyses are reporting of the pooled analyses with only studies with specific characteristics removed from the analyses. Please could the authors be more specific about the subgroup analyses performed and what sensitivity analyses have been performed with their justification?**

Reply: We have clarified that subgroup analysis was carried out based on characteristics of carotid stenosis symptoms (i.e., symptomatic, asymptomatic, mixed) in some analyses which had moderate heterogeneity (based on I^2^ = 30%-60% and Chi-square test (p<0.1). We additionally performed sensitivity analysis by removing studies affecting influence plot and excluding any studies that could not clearly identify recruitment periods. These justifications have been clarified in the Method section under ‘Statistical analyses’. The amended sentences are as follows.

*‘If there were moderate heterogeneity across studies, based on I^2^ 30%-60%, together with p value of Cochrane's Q <0.1 [22], we have attempted to seek possible factors using subgroups analysis. The subgroup analyses were determined by characteristics of carotid stenosis symptoms (i.e., symptomatic, asymptomatic, and mixed), while the sensitivity analyses were conducted by removing studies affecting the influence plot and any studies that could not clearly identify recruitment periods.*’ (p.8, lines 171-180)

**Minor comments:

1. The objectives in the abstract merely describe the methodology. Please describe the purpose of the meta-analysis based on the PICO question(s) i.e. investigate associations of age and sex on outcomes following CEA.**

Reply: Thank you for the comment. We have amended as you suggested. The objective has also amended in the main text in order to make a consistent statement between the abstract and the main text. The revised statement is as follows.

*‘investigate associations of age and gender on* *30 days post carotid endarterectomy (CEA) and up to 5 years post CEA stroke, death, and combined stroke and death’* (p.3, lines 47-49; p.5, lines 117-118)

**2. Please include the dates when the searches were last completed. They are mentioned in the abstract but not included in the manuscript that I can see (apologies if I've missed it).**

Reply: Thank you for the comment. For the first version of this manuscript, the years when the searches were last completed have been identified already. To make a consistent with the abstract section, we have added the further detail in this revised version. (p.6, line 124)

**3. I'm not sure what this means: "Hand searching which was proceeded via contacting expertise to further obtain published and unpublished studies." (lines 109-110) Please revise.**

Reply: We apologise to identify a mistake sentence which may be misleading. We have identified that hand searches were used to identify comprehensive searches, in addition to the original search from databases. Therefore, we have planned to do a hand searching by checking reference lists from the identified articles. We clarified it within Method section under ‘Search strategy’.

*‘To obtain comprehensive searches, hand searching was also carried out by checking reference lists from the identified articles.’* (p.6 lines 125-126)

**4. The order of reporting results seems strange. Whilst reporting risk of bias before presenting the results of the meta-analyses is usual, reporting the risk of publication bias and the GRADE assessments before presenting the actual results of the meta-analyses themselves seems strange. I think the more appropriate order would be to present the publication bias results after the results from the meta-analyses and then the GRADE assessments as the final** **part of the results.**

Reply: Thank you very much for the comment. We have re-ordered sections of result, publication bias, and GRADE assessment as you suggested.

**Review #2**

This study aimed to stratify by age and gender, the 30-day and 5-year stroke and mortality outcomes following carotid endarterectomy. The authors undertook an extensive search of the literature including 133 studies. They concluded that individuals over the age of 80 and those with asymptomatic disease had a higher mortality rate within 30 days of carotid endarterectomy. Additionally, women had a higher incidence of stroke within 30 days of carotid endarterectomy than men. While this study is of interesting and beneficial value, the article is, at times, difficult to read and to interpret with grammatical errors. Below are detailed reviews of each section and include suggestions to improve the manuscript.

ABSTRACT:
Objective to determine the outcomes of CEA however the authors only reported
the stroke and mortality rates. The word outcomes suggest that other factors were
included such as TIA and MI. Please reword this throughout the paper to make it clear to the reader that stroke and mortality were the only outcomes assessed.

Reply: The reviewer raised an important issue about study outcome that we should have been identified clearly. The specific outcomes of interests have been described as follows;

‘*investigate associations of age and gender on 30 days post carotid endarterectomy (CEA) and up to 5 years post CEA stroke, death, and combined stroke and death’* (p.3, lines 47-49)

We have also re-written as you suggested throughout the manuscript.

Line 47: First time CEA is mentioned, should be written out

Reply: The CEA has been revised to be full name, followed by its abbreviation as below.

*‘**30 days post carotid endarterectomy (CEA) and up to 5 years post CEA stroke, death, and combined stroke and death*’ (p.3, lines 47-49)

INTRODUCTION:
Line 82: Please provide a reference for this statement.

Reply: As requested, we have added the reference number 3 in the introduction (p.4, line 91).

*Added ref. ‘Naylor R, Rantner B, Ancetti S, de Borst GJ, De Carlo M, Halliday A. Editor's Choice - European Society for Vascular Surgery (ESVS) 2023 Clinical Practice Guidelines on the Management of Atherosclerotic Carotid and Vertebral Artery Disease. Eur J Vasc Endovasc Surg. 2023;65(1):7-111’.*

The second paragraph reads like a discussion proving an argument rather than setting the scene for the research undertaken. Consider rewording and restructuring.

Reply: We apologised for this confusing paragraph. We have now restructured in such paragraph (p.5, lines 94-118).

Objective – Please provide a definition for what is meant by short-term and long-term (i.e. 30 days post CEA and up to 5 years). This should also be clearly defined in the methods. The first time the reader reads this is in the results.

Reply: We have clarified that the *‘short term’* is defined as a period occurring within 30 days post CEA while *‘long term’* is defined as a period happening at up to 5 years post CEA. These details have been added in the Objective section and Method section as follows.

*In the objective section: ‘**…30 days post CEA (short-term) and up to 5 years post CEA (long-term) stroke, death, and combined stroke and death’* (p.5-6, lines 117-118)

*In the method section: ‘(i) studies investigating 30 days post CEA and/ or up to 5 years post CEA risk differences’* (p.6, line 135)

METHODS:
Line 140: Please explain what was meant by ‘Incomplete data was clarified by contacting an author of the study’. Was there a standard protocol followed? i.e. An email was sent to the author and if no response was heard within 2 weeks the results were not included.

Reply: Thanks for the comment. We have clarified that we followed the recommendation of Cochrane collaboration (Higgins JPT, Lasserson T, Chandler J, Tovey D, Churchill R. Methodological Expectations of Cochrane Intervention Reviews. Cochrane: London, 2016) to contact the author by an email if incomplete data was found. We have also determined the response length of time within 1 week. The revised one is as below.

*‘The corresponding author was sent an email to obtain additional information in case of incomplete data identified. If no response was received within a week, the result was excluded.’* (p.7 lines 159-161)

Statistical analysis: A random effect model should be used throughout rather than a fixed effect model.

Reply: We have re-analysed using random effects model throughout all analyses. We amended it within the section as follows;

We revised it within the following sections;

- Abstract: Result part (p.3-4, lines 62-70)
- Methods: Statistical analyses part (p.8, lines 169-170)
- Table 1 (p.10, line 202)
- Results: Effect of age on stroke, death, and combined stroke and death… (p.13-14, lines 215-235), Effect of gender on….(p.16-17, lines 261-277, 290-293)
- Figures 1-4
- S1 Fig. Other main results (S1_Fig_R1)
- S4 Table. Subgroup analyses (S4_Table_R1)
- S5 Table. Sensitivity analyses (S5_Table_R1)

RESULTS
Results section is difficult to read. If Odds Ratio and CI are reported in a table which is referenced then don’t include in the main body of the text as it is hard to follow. The effect of age and effect of gender are particularly difficult paragraphs to read, please restructure.

Reply: Thank you for the suggestion. We have removed the Odds and CI out of the main text accordingly. In addition, we have re-structured paragraphs as follows;

- Effect of age on stroke, death, and combined stroke and death risks following carotid endarterectomy

- Short-term effects (30 days post-surgery) (p.13-14, lines 215-235)

- Effect of gender on stroke, death, and combined stroke and death following carotid endarterectomy

- Short-term effects (30 days post-surgery) (p.16-17, lines 263-279)

- Long-term effects (up to 5 years post-surgery) (p.17, lines 292-295)

I hope this satisfied you.

Line 165: Split the studies into; age, gender, age and gender. This will make it easier for the reader

Reply: We have clarified this in the Results section (p.9, lines 191-193).

Line 186: Where does this number come from? First time referenced.

Reply: We apologized to identify the incorrect number. We have removed it. A corrected number of publications have been presented in the Results section (p.9, lines 191-193).

Table 2: Should define what their presenting symptoms were and carry out subgroup analyses accordingly as there are important differences in risk between different symptomatic indications. E.g. categorising symptomatic patients as; stroke, ocular event, cerebral TIA. Carrying out analysis by symptomatic or asymptomatic is too vague. This should be reflected in the discussion.

Reply: Thank you for the suggestions. We have clarified that we performed subgroup analyses based on characteristics of carotid stenosis symptoms (i.e., symptomatic, asymptomatic, and mixed) prior to performing surgery. As our review, most included studies have only identified that patients had either symptomatic carotid stenosis, asymptomatic carotid stenosis, or both without specifying the category of symptomatic patients as you mentioned. Thus, we then performed subgroup analyses based on the characteristics of carotid stenosis symptoms. I hope this amendment satisfied you.

DISCUSSION
Line 250: It is unclear where these percentages come from, is this from your data?

Reply: We have clarified that the percentages of mentioned cardiovascular risks were from the previous meta-analysis (ref. no.1). We have added further reference within the discussed sentence.

Line 269: Reference

Reply: As requested, we have added the reference (ref. no.29) within the discussed sentence.

Line 300: Reference the current Guideline

Reply: Reply: We have added the reference (ref. no 38) within the discussed sentence.

**Review #3**

A priori women and older people do worse in elective vascular surgery.

Although gender may be associated with worse outcomes, are there any clinical confounding variables that may contribute to this?

Reply: We have clarified that, in our study, female is associated with increased operative risks following CEA. In addition, there are other confounding factors that may contribute to the worse outcomes such as diabetes, dyslipidemia, and hypertension. Unfortunately, we could not perform subgroup or extract adjusted Odds Ratios in each included study as those studies’ objective were not primarily investigate association between age/ or gender and CEA on outcomes (stroke, death, combined stroke and death). For example, a study aimed to compare stroke event between two surgical procedures (CEA vs stent). In CEA, the stroke event was additionally identified in subgroup of age and gender. An adjusted Odd Ratio, if any, would only be available for the primary aim. Thus, we could only pick up event, non-event, and total number of patients for calculating Odd Ratio and pooled effect estimates for meta-analysis instead. However, we have added this important point as a limitation of the study. This has been clarified in the Strength and imitations section. The additional statement is as follow.

*‘Third, pooled effect estimates of this meta-analysis with unadjusted confounders are accounted as a significant limitation. As age and gender among most identified studies were not the main determinant of interest, we could not extract the adjusted effects. Thus, the meta-analysis finding should be interpreted with caution.’*

Hopefully, this responds you properly.

Please ask an English speaker e.g. Dr Howard to correct the English in the document please. It is substandard for a good scientific journal.

Reply: Thank you for the suggestion. We have improved the English writing throughout the manuscript again. Please note Dr. Howard and his team have scrutinized the whole document.

Line 82 please reference ESVS carotid guideline

Reply: We have added the reference of ESVS carotid guideline within the Strengths and limitations section.

*Added reference:* *‘Naylor R, Rantner B, Ancetti S, de Borst GJ, De Carlo M, Halliday A. Editor's Choice - European Society for Vascular Surgery (ESVS) 2023 Clinical Practice Guidelines on the Management of Atherosclerotic Carotid and Vertebral Artery Disease. Eur J Vasc Endovasc Surg. 2023;65(1):7-111.’*

Endpoints: one of the clinical concerns long term, is whether women with smaller arteries have a higher restenosis rate. Can the authors include this outcome please?

Reply: Thank you very much for your suggestion. We clarified that we aimed to focus only the stroke and death risks at 30-days post-CEA and long-term effect after CEA. In addition, the included studies of long-term effect of CEA rarely reported restenosis rate. Thus, we did not focus and analyse on this outcome.

It is interesting to see the peri-operative stroke risks are similar. This is reassuring in terms of efficacy of surgery.

Reply: Thank you very much for your suggestion. Of course, we agreed this finding reassured in terms of efficacy of surgery accordingly.

I think the odds ratios may be scientifically interesting but are too low (OR<2) to really influence clinical practice.

Reply: Thank you for the concern. We agreed with you that less OR (<2) is too low to impact clinical practice. Despite this, our study reported serious complication namely postoperative stroke and death risks. Odd ratio of such serious events even closer to 1 therefore can also be considered clinically significant.

Ref: Andrade C. Understanding relative risk, odds ratio, and related terms: as simple as it can get. J Clin Psychiatry. 2015;76(7):e857-61. doi: 10.4088/JCP.15f10150.

Older people with asymptomatic disease are not offered surgery – the ACST finding that older people have higher risks is the explanation, therefore this finding is redundant.

Reply: Thank you for the comment. We have clarified that, after year 2005, there are several studies reporting a controversial result with the ACST. These studies have suggested a reduction in operative risk for older people who had asymptomatic carotid stenosis (e.g., Glousman et al., 2020 and Stoner et al., 2006). Thus, we performed this meta-analysis to summarize the result within this context. However, our result still be similar with the previous meta-analysis and similar with the ACST. We have already discussed this point in the Discussion part.

It is no surprise that older people have more strokes and die more frequently than younger people. This finding does not require a meta-analysis.

Reply: Thank you for the comment. As mentioned above, there are accumulating studies reporting a conflicted result with the previous meta-analysis (Bond et al., 2005). Thus, we performed this meta-analysis to summarize the result within this context. However, our result still be similar with the previous meta-analysis. We have already discussed this point in the Discussion part.

There are several studies demonstrating the lower risk of stroke or death follow treatment of asymptomatic patients – e.g. ACST1 and 2, in comparison to symptomatic patients. Please comment.

Reply: Thank you for the comment. As you mentioned above, some studies (e.g., ACST-1 and 2 showed a lower risk of stroke in asymptomatic patients than those in symptomatic patients (e.g., ECST and NASCET). When we performed meta-analysis, the pooled effect estimates of asymptomatic female patients seemed to be more effect than those in symptomatic female patients.

I do not think that you can say that the risks are higher in asymptomatic females v symptomatic females as the 95%CI of the OR actually overlap. Please comment.

Reply: Thank you for the comment. We have clarified that symptomatic female patients had 48% and 26% significantly increased risk of stroke, and combined stroke and death than those with symptomatic male patient (S6 Appendix). Asymptomatic female patients had more increased risks as reflected by 51% and 50% significantly increased risk of stroke, and combined stroke and death than those with asymptomatic male patients (S6 Appendix). Although it is the OR directly computed between symptomatic and asymptomatic female patients, this can be indirectly inferred that more operative risks following CEA are found in asymptomatic patients. Hopefully, this satisfy you.

Similar comment for older patients. Please comment.

Reply: We have re-analysed the pooled effect estimates according to other reviewers’ comment and found that asymptomatic older patients had a 144% significantly increased risk of death than asymptomatic younger patients, while no association between symptomatic older patients and risk of death (S6 Appendix). This can be inferred that the increased mortality following CEA is associated with the older age especially those who had asymptomatic carotid stenosis.

Rothwell’s subgroup paper suggested that older symptomatic patients benefit more from CEA – would the author’s like to comment - i.e. procedural risks may be higher, but non-operated stroke risks are also higher.

Reply: Thank you for your comment. We have clarified that subgroup analysis of characteristics of carotid stenosis (i.e., symptomatic, asymptomatic) are performed based on heterogeneity. We have re-analysed the risk outcome based on other reviewers’ comment (please see the Figure of the main findings). After performing this, the subgroup analysis of age on stroke risk was not performed because there was no heterogeneity across the included studies.
